# Rapid evolution leads to differential population dynamics and top‐down control in resurrected *Daphnia* populations

**DOI:** 10.1111/eva.12567

**Published:** 2017-11-15

**Authors:** Eyerusalem Goitom, Laurens J. Kilsdonk, Kristien Brans, Mieke Jansen, Pieter Lemmens, Luc De Meester

**Affiliations:** ^1^ Laboratory of Aquatic Ecology, Evolution and Conservation KU Leuven Leuven Belgium

**Keywords:** eco‐evolutionary dynamics, ecosystem function, empirical dynamic modeling, local adaptation, population dynamics, resurrection ecology, top‐down control

## Abstract

There is growing evidence of rapid genetic adaptation of natural populations to environmental change, opening the perspective that evolutionary trait change may subsequently impact ecological processes such as population dynamics, community composition, and ecosystem functioning. To study such eco‐evolutionary feedbacks in natural populations, however, requires samples across time. Here, we capitalize on a resurrection ecology study that documented rapid and adaptive evolution in a natural population of the water flea *Daphnia magna* in response to strong changes in predation pressure by fish, and carry out a follow‐up mesocosm experiment to test whether the observed genetic changes influence population dynamics and top‐down control of phytoplankton. We inoculated populations of the water flea *D. magna* derived from three time periods of the same natural population known to have genetically adapted to changes in predation pressure in replicate mesocosms and monitored both *Daphnia* population densities and phytoplankton biomass in the presence and absence of fish. Our results revealed differences in population dynamics and top‐down control of algae between mesocosms harboring populations from the time period before, during, and after a peak in fish predation pressure caused by human fish stocking. The differences, however, deviated from our a priori expectations. An S‐map approach on time series revealed that the interactions between adults and juveniles strongly impacted the dynamics of populations and their top‐down control on algae in the mesocosms, and that the strength of these interactions was modulated by rapid evolution as it occurred in nature. Our study provides an example of an evolutionary response that fundamentally alters the processes structuring population dynamics and impacts ecosystem features.

## 1 INTRODUCTION

Ecological and evolutionary dynamics have long been considered as largely uncoupled and independent processes. More recently, it has become increasingly clear that both processes are strongly intertwined and can occur on the same time scales (Ellner, Geber, & Hairston, 2011; Hairston, Ellner, Geber, Yoshida, & Fox, 2005; Hendry, 2016; Schoener, 2011; Whitham et al., 2006). An increasing number of studies unequivocally demonstrate the existence of important feedbacks between evolutionary change and ecological dynamics (Bassar et al., 2010; Matthews et al., 2011; Pantel, Duvivier, & De Meester, 2015). For example, genetic diversity can profoundly alter population, community, and ecosystem characteristics (Crutsinger et al., 2006; Johnson, Vellend, & Stinchcombe, 2009; Whitham et al., 2006). Evolutionary trait change can mediate changes in population dynamics, community composition (Bassar et al., 2010; Matthews et al., 2011; Pantel et al., 2015; Terhorst, Lennon, & Lau, 2014; Urban et al., 2008), and ecosystem functions (Fussmann, Loreau, & Abrams, 2007). However, few of the studies so far report feedbacks of evolution that has been shown to have occurred in nature in a well‐defined time frame.

Predation by fish is an important determinant of variation in zooplankton and phytoplankton community characteristics in many lakes and ponds (Carpenter et al., 2001; Jeppesen et al., 2003). Fish are efficient predators that may not only affect the biomass, but also the qualitative characteristics of zooplankton communities, such as size distribution, species composition, and diversity (Declerck & De Meester, 2003; Lemmens, Declerck, Tuytens, Vanderstukken, & De Meester, 2017). Selective predation by fish can also have profound effects on population characteristics such as body size distribution, habitat use (Cousyn et al., 2001; De Meester, Weider, & Tollrian, 1995), and life‐history characteristics of species (Latta, Bakelar, Knapp, & Pfrender, 2007; Stoks, Govaert, Pauwels, Jansen, & De Meester, 2016). Adaptation to fish predation in zooplankton involves multiple life‐history (e.g., faster maturation at a smaller size, increased number of offspring, smaller offspring) and behavioral traits (e.g., diel vertical and horizontal migration; Boersma, Spaak, & De Meester, 1998; Stoks et al., 2016). These traits are expected to have a substantial impact on zooplankton population dynamics by reducing mortality in the presence of fish, as well as by their costs in terms of food intake, such as in the case of predator avoidance by diel horizontal or vertical migration, or differential allocation of energy into number and size of offspring (Walsh & Post, 2011). Changes in behavioral traits, life‐history characteristics, and population dynamics of zooplankton in the presence of fish are expected to also influence the phytoplankton community by altering top‐down control by zooplankton (Walsh, DeLong, Hanley, & Post, 2012). For example, a reduction in body size generally results in lower zooplankton grazing rates on phytoplankton (Gianuca, Pantel, & De Meester, 2016; Tessier, Leibold, & Tsao, 2000).

An increasing number of studies have shown that evolutionary responses to predation can impact predator–prey cycles and ecosystem characteristics. For example, the features of predator–prey cycles between rotifers and algae are profoundly altered by genetic variation in defense traits of the algae (Becks, Ellner, Jones, & Hairston, 2012; Fussmann, Ellner, & Hairston, 2003; Miller, Grand, Fondell, & Anthony, 2006; Yoshida, Jones, Ellner, Fussmann, & Hairston, 2003). Bassar et al. (2010) demonstrated that guppy populations adapted to different predation intensity change the features of small stream ecosystems by differentially lowering algal density and primary production, which results in altered nutrient cycles. In the water flea *Daphnia*, a set of studies quantifying eco‐evolutionary feedbacks in a lake food chain involving alewife predation has among others reported that *Daphnia* populations adapted to different levels of fish predation differentially impact algal biomass and dynamics (Post & Palkovacs, 2009; Post, Palkovacs, Schielke, & Dodson, 2008). A laboratory experiment with *Daphnia* populations obtained from different lakes that differ in zooplanktivorous fish predation intensity demonstrated that life‐history evolution in *Daphnia* resulted in divergence in the rate of population growth, which in turn altered consumer‐resource dynamics and ecosystem functions (Walsh et al., 2012). Adult anadromous alewives migrate into lakes during spring for spawning and migrate back to the ocean each autumn. In some lakes, however, alewife are present year‐round because they are land‐locked. *Daphnia* clones from lakes with anadromous alewives exhibited higher abundances and higher population growth, which resulted in consistently lower phytoplankton abundances compared to treatments with *Daphnia* from lakes with landlocked alewife populations or without alewife fish (Walsh et al., 2012).

While Walsh et al. (2012) documented a clear‐cut impact of evolution on population densities in *Daphnia* and associated increases in top‐down control of phytoplankton, in line with a priori expectations, the consequences of evolutionary change may not always be so straightforward. Given differential allocation into offspring and the impact of body size on grazing efficiency in zooplankton, the consequences of evolutionary change on population dynamics and ecosystem functions might depend on whether population dynamics are driven by resource limitation in juveniles or in adults (De Roos & Persson, 2013). In juvenile‐driven cycles, juveniles are the strongest competitors and can prevent adults from reproducing (Nilsson, Persson, & Van Kooten, 2010). Reproduction then only occurs once a whole cohort of juveniles matures. In case of adult‐driven cycles, adults are the strongest competitors and can prevent juveniles from maturing. The lack of new adults, while older ones die, eventually makes enough food available for some juveniles to mature (De Roos & Persson, 2013). *Daphnia* dynamics are still somewhat enigmatic in this respect, because the often observed high juvenile to adult biomass ratio suggests juvenile‐driven dynamics, but individual‐level laboratory experiments have shown that adults are stronger competitors than juveniles (De Roos, McCauley, Nisbet, Gurney, & Murdoch, 1997; Nisbet, McCauley, Gurney, Murdoch, & Wood, 2004). Fish predation pressure often results in a change in allocation toward the production of more but smaller offspring (Boersma et al., 1998; Reznick, Butler, & Rodd, 2001; Roff, 1993), and this may change the competitive ability and starvation resistance of the *Daphnia* juveniles (Tessier, Henry, Goulden, & Durand, 1983). As a result, evolutionary change in response to an increase in predation pressure might change competitive interactions between adults and juveniles that drive population dynamics.

In earlier resurrection ecology studies, Cousyn et al. (2001) and Stoks et al. (2016) have reported rapid genetic adaptation of life‐history and behavioral traits in a natural population of the water flea *D. magna* in response to changes in fish predation pressure that occurred over a time period of 16 years. Given the substantial changes in 13 of the 14 studied trait values that were reported in Stoks et al. (2016) combined with the well‐documented high grazing pressure on algae that is exerted by large‐bodied *Daphnia* such as *D. magna* (Carpenter, Cottingham, & Schindler, 1992; Gianuca et al., 2016; Lampert & Sommer, 2007; Verreydt et al., 2012), it is our hypothesis that these evolutionary changes likely influence population dynamics of the *Daphnia* themselves as well as algal dynamics and top‐down control. We here took the opportunity to test the hypothesis of a feedback of evolution as it occurred in nature on a key ecosystem function in an outdoor mesocosm experiment in which we quantified *Daphnia* population densities and phytoplankton biomass over time in mesocosms inoculated with a representative set of clones of the three resurrected populations studied by Cousyn et al. (2001) and Stoks et al. (2016). These populations strongly differ in life‐history and behavioral traits (Stoks et al., 2016) and were here inoculated in mesocosms that did or did not contain fish. The presence and absence of fish provide very different selection pressures. For instance, *Daphnia* might adapt to the presence of visual predators such as fish by evolving a smaller body size (Stoks et al., 2016). In the absence of predation, however, *Daphnia* populations can reach a higher biomass, which increases food shortage, and thereby might select for larger *Daphnia* that produce larger‐sized offspring with more reserves (Guisande & Gliwicz, 1992). Hence, we expected that in the treatment without fish, the *Daphnia* population resurrected from the period prior to fish stocking would be able to attain the highest densities and exert the strongest top‐down control on algae. In the presence of fish in the mesocosms, we expected that the population resurrected from the period with highest fish stocking would reach the highest densities because this population is adapted to coexist with fish and thus better protected from fish predation. As a result, we expected this population to exert the strongest control on algal biomass in the mesocosms with fish.

The main objective of our study was to test the feedback of evolution as it occurred in nature on an ecosystem function. Our study was therefore designed to test whether different populations established through a resurrection ecology study differed in population densities and top‐down control of algae in a common gardening mesocosm experiment (Matthews et al., 2011). Our results do show pronounced differences among the populations, but the observed pattern was more complicated than our straightforward expectations. We therefore also engaged in an effort to elucidate the mechanisms underlying the observed differences between the populations that were resurrected from a layered egg bank of a single pond and document evolution as it occurred in a single population over a period of approximately 16 years.

## 2 MATERIAL AND METHODS

### 2.1 *Daphnia* populations used in the experiment

The *D. magna* clones used in the experiment were obtained from sediment cores from a relatively small (8.7 ha), shallow pond that was constructed in 1970 for the purpose of fish culture (“Oud‐Heverlee Zuid,” Belgium 50°50′22.16″N, 4°39′18.16″E). This pond has a well‐documented fish‐stocking history over 30 years of its existence (see Cousyn et al., 2001). No fish stocking occurred in the period 1970–1972, while large numbers of planktivorous fish were stocked (>250 kg/ha) from 1976 to 1979. Thereafter, the stocking decreased and completely stopped in 1993. We can therefore distinguish three main periods with regard to fish predation intensity in the history of the pond: a period corresponding to the first years (1970–1972) after the pond was dug when no fish were present (here called “pre‐fish period”), a period of high fish predation pressure (between 1976 and 1979; called “high‐fish period”), and a period of relaxed fish predation pressure from 1988 to 1990; called “reduced‐fish period; (see also Stoks et al., 2016). There was only a low level of genetic differentiation in neutral microsatellite markers between the three populations separated in time (Cousyn et al., 2001), supporting the view that they represent one single continuous population that showed strong adaptive evolution.

Ephippia of *D. magna* clones were collected from three depth layers of a sediment core, corresponding to the pre‐fish, high‐fish, and reduced‐fish period (Cousyn et al., 2001). The sediment sampling and hatching were carried out as part of a previous resurrection ecology study (Stoks et al., 2016). In the laboratory, ephippia were exposed to optimal hatching stimuli (16‐hr light/8‐hr dark; 20°C, fresh medium) and twelve clonal lineages from each fish‐stocking period were obtained and kept in the laboratory as clonal cohorts for several years before the experiment. During stock cultures, the clones were maintained at low food to keep them at low densities. Estimated population sizes (0.5‐L jars) were less than 20 individuals; most individuals carried one or two eggs maximum, and average life span is estimated to be more than 3 months under those conditions (Luc De Meester personal observations). In this way, the turnover in individuals per year is very low (estimated to be less than 200 individuals per year) so that the probability of mutations impacting the genotypic trait values of individual clones is low, even over a period of 10 years. The clones used in the present experiment were the same as used in Stoks et al. (2016), except for a contamination problem involving a few lineages (see Supporting Information  SI1).

In preparation for the experiment, we started up four independent, replicate cultures of all 36 clones individually (12 clones per time period × 3 time periods) under standardized conditions in a climate room (20 ± 1°C with a 16L:8D photoperiod). Half of the culture medium (dechlorinated tap water) was renewed daily, and the animals were fed fresh green algae (*Acutodesmus obliquus*, 1 × 10^5^ cells/ml). Interference from maternal effects was minimized by growing the animals for two generations under those standard conditions prior to the mesocosm experiment. After the release of the second clutch of the second generation, we randomly selected 10 juveniles per clone and per replicate as the basis for the inoculum of the mesocosms. Per clone and replicate five individuals were assigned to the Predation treatment, the other five to the Control treatment. Those five individuals were grown together in a 500‐ml jar until release of the second clutch. In total, our setup involved 3 time periods × 12 clones × 2 treatment groups × 4 replicates = 288 culture units. From the second clutch, we randomly selected 12 juveniles per clone, layer, replicate, and treatment and combined them per layer to a population that was inoculated in a mesocosm. Each mesocosm thus received 144 individuals representing 12 genotypes of one population (pre‐fish, high‐fish, or reduced‐fish), each represented by 12 individuals. Each replicate of a population (pre‐fish, high‐fish, or reduced‐fish) × treatment (presence or absence of predation) combination received an inoculum of animals that had been kept in separate culture for at least two generations. In this way, significant differences between mesocosms inoculated by different populations can be attributed to genetic differences among the populations rather than to maternal effects or effects of physiological acclimation. All animals were inoculated in the mesocosms when they were 24–48 hr old.

### 2.2 Mesocosms experiment

Twenty‐four cylindrical polyethylene 200 L mesocosms (three populations x two treatments x four replicates) were placed in an open grass field at the outdoor experimental area of the laboratory of Aquatic Ecology, Evolution and Conservation (ARENA) in Heverlee, Belgium. All mesocosms contained a fish cage made of 5‐mm plastic mesh netting and representing one‐third of the mesocosm volume, leaving a refuge of approximately 10 cm at the bottom and along the sides of the mesocosm. These refuges are similar to the ones used by zooplankton to avoid fish predation through horizontal and diel vertical migration. Each mesocosm was covered with mosquito netting (1.2 mm mesh size) to prevent mosquitoes and other insects from entering into the mesocosms. On July 1, 2014, the mesocosms were filled with 180 L of tap water, three liter of filtered (64 μm mesh size) water from a natural pond, and 10 ml of an *Acutodesmus obliquus* green algae suspension (1 × 10^8^ cells/ml). The addition of pond water and the *Acutodesmus* inoculum was intended to stimulate the growth of phytoplankton. After twenty‐one days, the mesocosms were randomly assigned to the Control (*n* = 12) and Predation treatment (*n* = 12), and within each of these two treatments to one of the three *Daphnia* population treatments (pre‐fish, high‐fish, and reduced‐fish). On that day (day 0 of the experiment), each mesocosm received 144 juvenile *Daphnia* representing independently cultured representatives of all clones from a given population (see above). Sixteen days after inoculating the mesocosms with *Daphnia* (i.e., day 16 of the experiment; slightly more than one parthenogenetic *Daphnia* generation at 20°C, ensuring that the inoculated individuals had reproduced), we added one three‐spined stickleback (*Gasterosteus aculeatus*) of a standard body length of 5 cm to the cages of the Predation mesocosms. Every six days, the fishes were taken out and redistributed using a randomization scheme to eliminate any possible biases that might arise because of differential activity among individual fishes.

### 2.3 Abiotic and biotic variables

We aimed for a regular increase in nutrient concentrations in the mesocosms to prevent nutrient limitation and promote the growth of phytoplankton, thereby challenging the capacity of the *Daphnia* population to achieve continued top‐down control of the phytoplankton. In this way, we also buffered for the increase in nutrients imposed by excretion of the fish in the Predation treatment. To this end, water samples were taken every three days from each mesocosm, pooled per treatment (Predation and Control treatment), and immediately analyzed for total nitrogen (TN; two missing values on days 30 and 39) and total phosphorus (TP) using a HACH spectrometer. For TP, we aimed a weekly increase of 0.2 mg/L starting from day 31. Based on the measured TP concentration, we calculated the amount of P that was needed to obtain an increase of 0.2 mg TP/L per week in both the Predation and Control mesocosms. As the Predation mesocosms met the required increase in TP spontaneously (due to the excretion of P by the fish), we only added phosphorus (as KH_2_PO_4_) in mesocosms of the Control treatment. Every second time that we added P, we also added micronutrients (Na_2_EDTA, FeCl_3_, CuSO_4_, ZnSO_4_, CoCl_2_, MnCl_2_, Na_2_M_O_O_4_, and H_3_BO_3_). To achieve a reasonable ratio between TP and TN concentrations, we added on two occasions nitrogen (as NaNO_3_) in both the Predation and Control mesocosms. Because of the procedure to only add micronutrients every second time we added phosphorus and the spontaneous increase in TP in the mesocosms of the Predation treatment, we did not add micronutrients in mesocosms of the Predation treatment. This might be the cause for our observation that the Predation mesocosms experienced less pronounced algal blooms than the Control mesocosms (see further). Figure SI2 in Supporting Information shows the changes in average total phosphorus and total nitrogen concentrations in the Predation and Control mesocosms as measured every three days along with the changes in temperature during the course of the experiment. The experiment lasted for 70 days.

From day 15 onwards, all mesocosms were intensively monitored until the end of the experiment. Water temperature was measured in each mesocosm every three days using a HACH multimeter. The concentration of *in vivo* chlorophyll *a* was used as a measure of phytoplankton biomass and was monitored daily (one missing value on day 59) with a handheld fluorometer (AquaFluor, Turner Designs, Sunnyvale, CA, USA). The *Daphnia* population was sampled in each mesocosm every three days, except for the last sampling, which was delayed by one day (cf. day 70 instead of day 69). The *Daphnia* were sampled by taking a water sample (2 L) after gently mixing the water in the mesoscosm with a tube sampler. The two‐liter water sample was taken using a beaker and filtered over a 64 μm mesh size plankton gauze. Zooplankton samples were preserved in 4% formaldehyde. The number of adult and juvenile *Daphnia magna* individuals was determined in each sample by counting a minimum of 300 individuals from each sample using a stereomicroscope (Olympus ZS X 12). The counts were extrapolated to the total volume of the sample and transformed to abundances per liter (number of individuals/liter). *Daphnia* adults and juveniles were differentiated based on the length of the first abdominal process, which is clearly elongated in adult compared to immature females to be able to close the brood pouch (Benzie, 2005).

### 2.4 Data analysis

**Figure 1:**
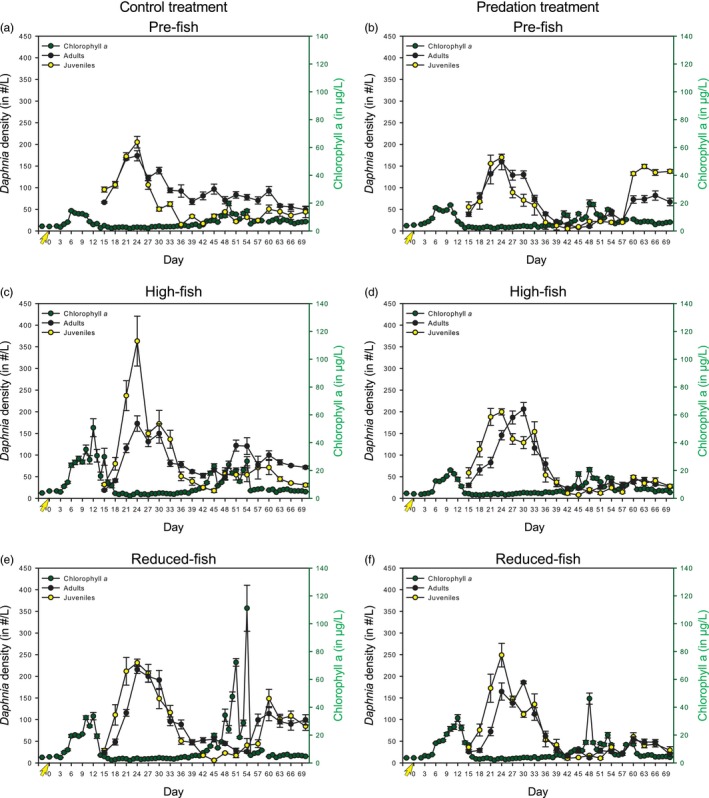
Average chlorophyll *a*, adult *Daphnia* and juvenile *Daphnia* abundance over the four replicates for each population (Pre‐fish, High‐fish, and Reduced‐fish) at each time point in the Predation and Control treatment. Error bars denote standard error. Yellow arrows denote the inoculation with juvenile *Daphnia* (0.8 individuals per liter)

As a first test of differences among populations (categorical: pre‐fish, high‐fish, and reduced‐fish) in *Daphnia* abundance and chlorophyll *a* concentration, we carried out a repeated‐measures linear mixed‐effect model (pairing data according to date) using the “nlme” and “car” packages in R to compute approximate *F*‐test statistics and *p*‐values for fixed effects (R Development Core Team, 2016). For each variable, population was entered as a fixed effect and replicate populations were included as a random effect. We applied the restricted maximum‐likelihood estimation method (REML). We analyzed the data of the Control and Predation treatment separately because of the difference in micronutrient addition during the experiment (see abiotic and biotic variables). Tukey Post hoc tests (“multcomp” package in R) were used to test for significant differences among specific populations in case of a significant main effect of population. We used (daily) chlorophyll *a* data and *Daphnia* abundance each three days from day 15 onwards. Chlorophyll *a* measurements before day 15 were part of the acclimation period and not used in the analyses (they are, however, plotted for clarification in Figure 1 and Fig. SI3).

The repeated‐measures linear mixed‐effect model we carried out is able to find some of the differences in population dynamics, but only when replicates behave in a synchronized and linear way. In Table SI1 (see Supporting Information SI2), we show that for 15 of the 18 population × treatment × variable combinations the dynamics are in fact nonlinear (i.e., theta > 0) and we also find a decay in forecast skill for long term forecasts, which is a characteristic of nonlinear systems. In the Supporting Information, we therefore also provide a test for population differences based on simplex projections that do not assume linearity and synchrony, as additional support for differences in population dynamics. Simplex projections are an empirical dynamic modeling (EDM) technique (Deyle, Maher, Hernandez, Basu, & Sugihara, 2016; Deyle, May, Munch, & Sugihara, 2016; Sugihara, 1994; Sugihara et al., 2012; Sugihara & May, 1990; see Supporting Information SI2). In the simplex projection‐based test for population differences, we compared the forecast skill of simplex projections using training and testing sets (replicate time series) from the same or from different populations (see Supporting Information SI2).

To explore the mechanisms underlying the differences in population densities and top‐down control among populations, we examined the interactions between population densities of adult *Daphnia*, population densities of juvenile *Daphnia*, and phytoplankton biomass. Phytoplankton biomass, *Daphnia* adult abundance, and *Daphnia* juvenile abundance together form a dynamic system in each mesocosm. They are (potentially) all affecting each other, and these interactions can vary along a range of strengths depending on the state of the system. The interaction strength can, for instance, show us if adults are suppressing juveniles or vice versa, and thereby provide a powerful way to distinguish adult‐driven dynamics from juvenile‐driven dynamics. Furthermore, the values of the interaction strengths between these three variables can differ among populations if genetic differences in life‐history or behavioral traits between these populations cause differences in the strengths and directions of interactions between juveniles, adults, and phytoplankton biomass. To estimate the interaction strengths, we used S‐maps on the time series (Sugihara, 1994) as described in Deyle, May et al. (2016). S‐map is another empirical dynamic modeling (EDM) technique that has been used to detect nonlinearity in dynamic systems (Sugihara & May, 1990) and make forecasts (Sugihara, 1994) of nonlinear responses within time series. The S‐map method uses a locally weighted linear regression scheme, such that based on the state of the system, different regression coefficients are used for each forecast. These regression coefficients become estimates of interaction strength when making forecasts one time step into the future, using a multivariate embedding (i.e., a set of variables used as predictors in the regression and to determine the state of the system), which contains different variables from that system. More precisely, these interaction strengths are dynamic forecasts of the effect one variable has on another variable one time step later (Deyle, May, et al., 2016). We produced S‐maps based on normalized time series data from each mesocosm. Libraries were created for each treatment and each population separately based on data from all four replicates combined. Combining replicate time series was carried out following Hsieh, Anderson, and Sugihara (2007) and Clark et al. (2015). We only used chlorophyll *a* data from the days at which also the *Daphnia* densities were quantified, that is, every third day (except for the last measurement, which was delayed by one day). We expressed time (t) in days, and thus, S‐map forecasts were made for t + 3. S‐map coefficients were calculated to estimate the effect of each of the three variables (chlorophyll *a* concentration, adult *Daphnia* densities, and juvenile *Daphnia* densities) on each other and on themselves. Each S‐map used all three variables for the embedding (see Supporting Information SI2). Before interpreting the S‐map, we used convergent cross mapping (CCM) and associated null tests with surrogate time series (see Supporting Information SI2) to test whether the interactions are significant (Deyle, Maher, et al., 2016; Sugihara et al., 2012). CCM tests were carried out for each population and treatment separately.

Given that interactions between juveniles and adults and its effects on top‐down control can be mediated by competition and thus be influenced by food levels, we tested for correlations between estimated interaction strengths and phytoplankton biomass using linear and quantile regression. Note that these tests show patterns in the model estimates of the interaction strengths rather than in the real interaction strengths. The forecast skills of the models were evaluated by the mean absolute error (MAE) and the correlation (ρ) between observations and model predictions. The degree to which patterns in model estimates reflect patterns in real interaction strengths can be derived from the skill of the model forecasts (Supporting Information SI2, Table SI1).

All analyses and calculations were carried out in R v3.3.1 (R Development Core Team) using multiple functions from the R package rEDM developed by Ye, Clark, Deyele, Keyes, and Sugihara (2016) with additional information from Deyle, Maher et al. (2016) and Deyle, May et al. (2016). In all analyses, the data from the Control and Predation treatment were interpreted independently as both treatments received different concentrations of micronutrients throughout the duration of the experiment (only the Control mesocosms received micronutrients along with additions of phosphorus; the Predation mesocosms did not because TP increased spontaneously in these mesocosms; see abiotic and biotic variables).

## 3 RESULTS

The overall dynamics were quite similar across mesocosms (Figure 1 and Fig. SI3). During an initial phase (day 15 till approx. day 35), there was first a strong increase in the density of *Daphnia* adults and juveniles followed by a pronounced decrease. In between approximately day 40 to approximately day 55, densities of juveniles were very low and chlorophyll *a* levels tended to increase in many of the mesocosms. This increase in phytoplankton biomass was very strong in some mesocosms, whereas in others, there were only moderate fluctuations. From approximately day 55 onwards, the number of juveniles in most mesocosms started to increase and chlorophyll *a* levels were suppressed.

### 3.1 Population differences

**Figure 2:**
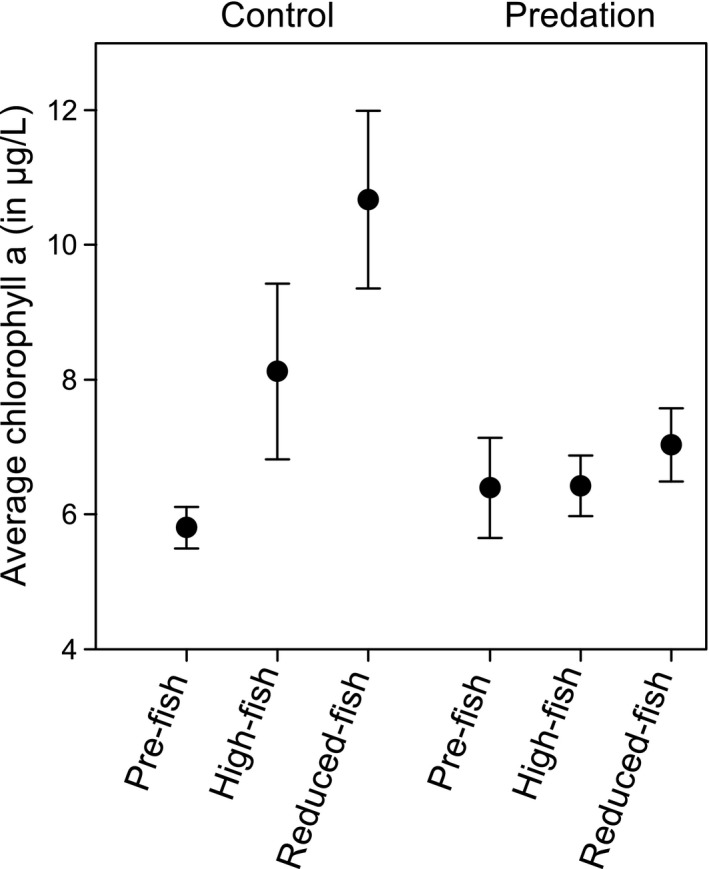
The average chlorophyll *a* concentration for the three populations (Pre‐fish, High‐fish, and Reduced‐fish) in the Predation and Control treatment. Error bars denote one standard error

In the Control treatment, linear mixed‐effect model revealed significant differences in chlorophyll *a* levels and juvenile abundances among *Daphnia* populations (Figures 1a, c & e and 2, Table 1A). The reduced‐fish population mesocosms had a significantly higher chlorophyll *a* concentration compared to the pre‐fish population mesocosms (post hoc Tukey test, Table 1A). The high‐fish and reduced‐fish populations differed significantly in juvenile *Daphnia* abundances from the pre‐fish population (post hoc Tukey test, Table 1A). In the Predation treatment, the linear mixed‐effect model showed no significant differences in chlorophyll *a* levels and *Daphnia* abundance among the three populations (Figure 2, Table 1B). In the Control treatment, simplex projections of both the phytoplankton biomass and the juvenile *Daphnia* abundances in the reduced‐fish mesocosms were significantly better forecasted using other time series from reduced‐fish mesocosms as library (i.e., training set) than when using one of the other two populations, that is, pre‐fish or high‐fish (Table 1A, Supporting Information SI2, Fig. SI8). Thus, reduced‐fish populations had dynamics in phytoplankton biomass and juvenile dynamics not present in the other two populations (also see Supporting Information SI2). In accordance with the linear mixed‐effect model, we found no significant differences among populations in adult *Daphnia* abundances in the Control treatment (Table 1A). In the Predation treatment, simplex projections revealed differences in phytoplankton biomass between the reduced‐fish population and the pre‐fish and high‐fish populations (Table 1B, Supporting Information SI2, Fig. SI8). For Adult *Daphnia* abundances, simplex projections identified with statistical significance dynamics in the high‐fish population not present in the pre‐fish population (Table 1B, Supporting Information SI2, Fig. SI8). For the juvenile dynamics, simplex projections identified dynamics in the pre‐fish population time series not present in the high‐fish and reduced‐fish populations (Table 1B, Supporting Information SI2, Fig. SI8).

**Table 1:** Results on population differences in chlorophyll *a* concentration*,* adult *Daphnia* abundance, and juvenile *Daphnia* abundance for the pre‐fish, high‐fish, and reduced‐fish populations in the (A) Control treatment and (B) Predation treatment using linear mixed‐effect models structured with repeated measures and supplemented by Tukey post hoc tests and using simplex projections

	Linear mixed‐effect model			Tukey post hoc test			Simplex projections			
	*df*	*F*	*p*							
(A) Control treatment										
Chlorophyll *a* concentration					High‐fish	Reduced‐fish		Pre‐fish	High‐fish	Reduced‐fish
Intercept	1	126.24	<.01	Pre‐fish	X	✓**.001**	Pre‐fish		X	✓ (4)
*Daphnia* population	2	6.42	**<.01**	High‐fish		X	High‐fish	X		✓ (1)
Day	54	4.60	**<.01**				Reduced‐fish	X	X	
Adult *Daphnia* abundance					High‐fish	Reduced‐fish		Pre‐fish	High‐fish	Reduced‐fish
Intercept	1	364.63	<.01	Pre‐fish	X	X	Pre‐fish		X	X
*Daphnia* population	2	0.27	.76	High‐fish		X	High‐fish	X		X
Day	18	9.53	**<.01**				Reduced‐fish	X	X	
Juvenile *Daphnia* abundance					High‐fish	Reduced‐fish		Pre‐fish	High‐fish	Reduced‐fish
Intercept	1	284.05	<.01	Pre‐fish	**✓.023**	**✓.023**	Pre‐fish		X	✓ (4)
*Daphnia* population	2	4.54	**.01**	High‐fish		X	High‐fish	X		✓ (5)
Day	18	11.75	**<.01**				Reduced‐fish	X	X	
(B) Predation treatment										
Chlorophyll *a* concentration					High‐fish	Reduced‐fish		Pre‐fish	High‐fish	Reduced‐fish
Intercept	1	263.37	<.01	Pre‐fish	X	X	Pre‐fish		X	✓ (2)
*Daphnia* population	2	1.10	.33	High‐fish		X	High‐fish	X		✓ (5)
Day	54	10.47	**<.01**				Reduced‐fish	X	X	
Adult *Daphnia* abundance					High‐fish	Reduced‐fish		Pre‐fish	High‐fish	Reduced‐fish
Intercept	1	152.79	<.01	Pre‐fish	X	X	Pre‐fish		✓ (3)	X
*Daphnia* population	2	1.14	.32	High‐fish		X	High‐fish	X		X
Day	18	20.27	**<.01**				Reduced‐fish	X	X	
Juvenile *Daphnia* abundance					High‐fish	Reduced‐fish		Pre‐fish	High‐fish	Reduced‐fish
Intercept	1	307.16	<.01	Pre‐fish	X	X	Pre‐fish		✓ (4)	✓ (4)
*Daphnia* population	2	0.05	.95	High‐fish		X	High‐fish	✓ (1)		X
Day	18	12.21	**<.01**				Reduced‐fish	✓ (1)	X	

Symbol “✓” indicates significance, and symbol “X” indicates nonsignificance. The number between brackets for the report on the simplex projections indicates the number of forecast time step lengths, of the 5 tested, in which the row population could significantly better predict itself than the column population could (see Supporting Information SI2 for more details on the results). Significant *p*‐values are shown in bold.

### 3.2 Interactions underlying the dynamics of juvenile and adult *Daphnia*

**Figure 3:**
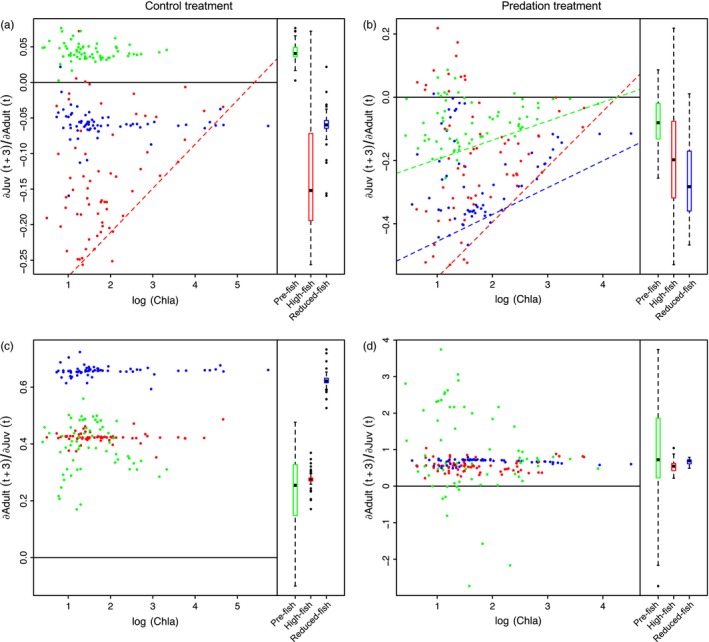
The effect of *Daphnia* adults on *Daphnia* juveniles [∂Juv(t + 3)/∂Adult(t)] (a,b) and the effect of juveniles on adults [∂Juv(t + 3)/∂Adult(t)] (c,d) as a function of phytoplankton biomass (log(Chla)) for each population separately (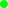 Pre‐fish, 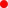 High‐fish, and 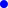 Reduced‐fish) in the absence (a,c) and presence (b,d) of predation. Simple linear regressions (dashed lines) show significant (*p* < .005, see text) 0.05 quantile regressions between estimated interaction strengths and the log(Chla) for each population. Boxplots show the distribution of estimated interaction strengths for the three populations. The bottom and top of the box show the lower and upper quartiles, the band in between them shows the median; whiskers show the minimum and maximum (excluding outliers), and circles show the outliers. Outliers are values more than 1.5 times the length of interquartile range larger than the upper quartile or smaller than the lower quartile. The S‐map estimated interaction strengths are in normalized units. The solid line shows the line of no effect

In the Control treatment, CCM tests showed significant effects of the density of adult *Daphnia* on the number of juvenile *Daphnia* in the high‐fish and reduced‐fish populations, but not in the pre‐fish population (Supporting Information SI2, Fig. SI9). S‐maps indicated this effect was on average negative (Figure 3a). The estimated strength of this interaction became smaller at high phytoplankton biomasses for the high‐fish population (Figure 3a, 0.05 quantile regression: *t* = 2.96869, *p* < .01), while we did not observed a significant relation between the estimated interaction strength and phytoplankton biomass for the reduced‐fish population. In the Predation treatment, CCM tests identified significant effects of the density of adult *Daphnia* on the number of juveniles in all three populations (Supporting Information SI2, Fig. SI9). In all three populations S‐maps indicated this effect was on average negative and limited in strength at high phytoplankton biomasses (Figure 3b, 0.05 quantile regression, pre‐fish: *t* = 3.78178, *p* < .001; high‐fish: *t* = 6.5038, *p* < .001; reduced‐fish: *t* = 3.53450, *p* < .001).

In the Control treatment, CCM tests revealed that the population dynamics of *Daphnia* juveniles had a significant effect on the number of adults in all populations (Supporting Information SI2, Fig. SI9). S‐maps indicated this effect was positive on average in all populations (Figure 3c). This effect was not associated with phytoplankton biomass in any of the populations (see Figure 3c). In the Predation treatment, CCM tests indicated juveniles had a significant effect on the number of *Daphnia* adults in all three populations (Supporting Information SI2, Fig. SI9). The S‐map estimates of this interaction were on average positive in all three populations (Figure 3d). In the high‐fish and reduced‐fish population, there was little variation in the extent of this effect, while in the pre‐fish population it was highly variable (Figure 3d). There was no clear relation between the estimated effect of juvenile on adult *Daphnia* density and phytoplankton biomass (Figure 3d).

The estimated effect of adult *Daphnia* density on adult *Daphnia* was positive in both the Predation and Control treatment (Fig. SI4 a & b). In the Control treatment, this positive effect was considerably higher for the pre‐fish population than for the other two populations (Fig. SI4 a). We did not observe this difference in the Predation treatment, but here the pre‐fish population showed larger variability in the estimated impact of adults on adults than the other two populations (Fig. SI4 b). The effects of juvenile *Daphnia* on juveniles were estimated to be always positive in both Predation and Control treatment (Fig. SI4 c & d).

In the Predation treatment, the CCM tests identified a significant effect of phytoplankton biomass on adults, which the S‐maps estimated was negative in all populations (Supporting Information SI2, Figs SI9, SI5). CCM tests also identified a significant effect of phytoplankton biomass on juveniles in the pre‐fish and high‐fish populations, which with S‐maps was also estimated to be negative on average in both populations (Supporting Information SI2, Figs SI9, SI5).

### 3.3 Interactions underlying the dynamics of chlorophyll *a*

**Figure 4:**
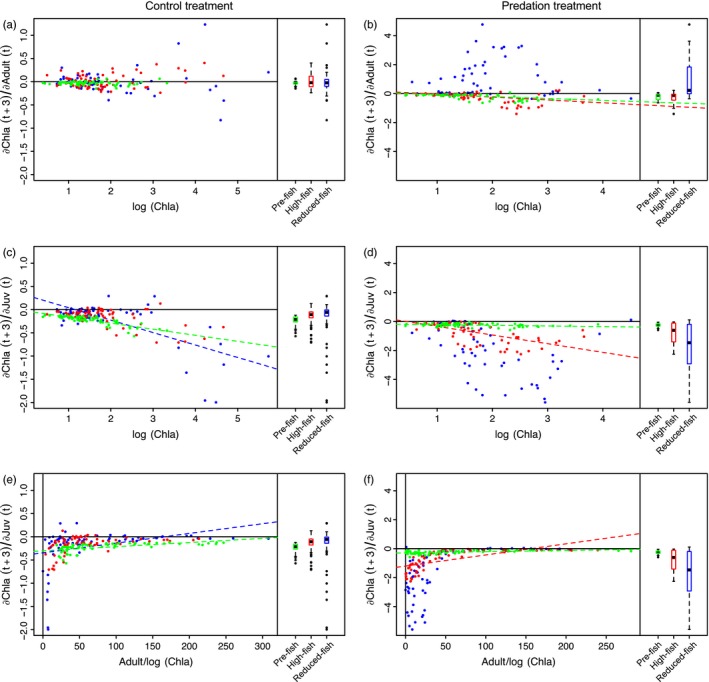
The effect of adults on phytoplankton [∂Chla(t + 3)/∂Adult(t)] (a,b) and the effect of juveniles on phytoplankton [∂Chla(t + 3)/∂Juv(t) (c,d) as a function of the phytoplankton biomass (log(Chla)) in each population in the absence (a,c) and presence of predation (b,d). (e,f) The effect of juveniles on phytoplankton [∂Chla(t + 3)/∂Juv(t)] as a function of the ratio Adult : log(Chla) in the absence (e) and presence of predation (f). The three populations are each time shown as 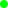 Pre‐fish, 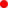 High‐fish, and 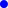 Reduced‐fish. Simple linear regressions (dashed lines) between the S‐map estimated interaction strengths and the log(Chla) in the Pre‐fish (green), High‐fish (red), and Reduced‐fish (blue) populations all had significant slopes (*p* < .05, see text). Boxplots show the distribution of estimated interaction strengths for the three populations. The bottom and top of the box show the lower and upper quartiles, the band in between them shows the median; whiskers show the minimum and maximum (excluding outliers), and circles show the outliers. Outliers are values more than 1.5 times the length of interquartile range larger than the upper quartile or smaller than the lower quartile. The S‐map estimated interaction strengths are in normalized units. The solid line shows the line of no effect

**Figure 5:**
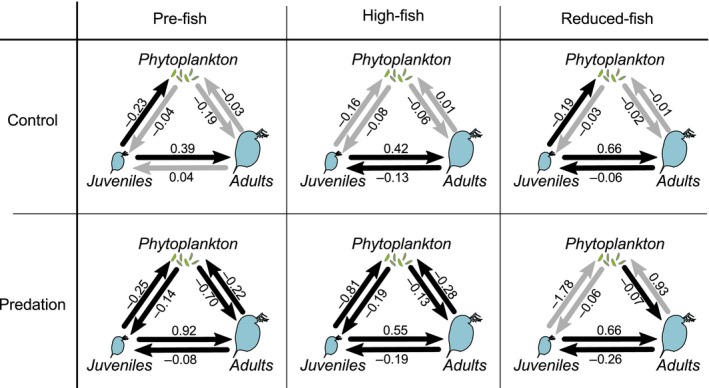
Interaction network for each population in both treatments (Predation and Control). Networks are based on cross map skills (ρ_ccm_) and average S‐map estimates of interaction strength. Black arrows show interactions for which the ρ_ccm_ was significantly larger than the surrogate time series based null distributions of ρ_ccm_. Numbers next to the arrows indicate the average interaction strengths as was estimated using S‐maps

Convergent cross mapping tests revealed no significant (Supporting Information SI2, Fig. SI9) effect of the density of adult *Daphnia* on phytoplankton biomass in all three populations in the Control treatment (Figure 4a), whereas the effect of adults on phytoplankton biomass was significant for the pre‐fish and high‐fish populations in the Predation treatment and estimated to be on average negative using S‐maps (Figures 4b and 5, Fig. SI9). For both populations, this negative effect was stronger at higher phytoplankton biomass (Figure 4b).

In the Control treatment, the effect of juveniles on phytoplankton biomass was significant and had on average negative S‐map estimates in the pre‐fish and reduced‐fish populations (Figures 4c, 5 and Fig. SI9). In the Predation treatment, the effect of juveniles on phytoplankton was significant in the pre‐fish and high‐fish populations (Figures 4c–f and 5 and Fig. SI9). In all cases, simple linear regressions revealed that the estimated effect of juveniles on phytoplankton was stronger at high than at low phytoplankton concentrations (Table SI2) and at low rather than high ratios of *Daphnia* over phytoplankton biomass (Figure 4c–f, Table SI2). In all cases, the S‐map estimated negative effect of *Daphnia* juveniles on phytoplankton biomass was (much) stronger than the estimated effect of *Daphnia* adults on phytoplankton (Figure 4). The negative effect of juveniles on phytoplankton biomass was large when the densities of adults and juveniles were low (Figs SI6 and SI7).

## 4 DISCUSSION

### 4.1 Population differences

At first glance, we observed a rather repeatable pattern in all mesocosms, which reflects observations in many other studies on *Daphnia* dynamics (Nelson, McCauley, & Wrona, 2005; Walsh et al., 2012), with a rapid population growth at the start of the experiment that apparently results in an overshooting of carrying capacity and is followed by a pronounced decline in population densities. During this initial phase, the *Daphnia* rapidly start to control phytoplankton growth, and chlorophyll *a* levels remain low in all mesocosms. At the end of this phase of decline in *Daphnia* densities, and in general, when the number of juveniles becomes very low, there is in many mesocosms a quite pronounced increase in chlorophyll *a* biomass, reflecting that the top‐down control by *Daphnia* is not effective anymore. After a period of increased phytoplankton biomass, the *Daphnia* densities start to slightly increase again, and the *Daphnia* again exert top‐down control over the algae. As a result, the mesocosms in our 70 days experiment only showed a temporary increase in chlorophyll *a*, during the period between 40 and 55 days. The intensity of the resulting phytoplankton bloom differed strongly among mesocosms. *Daphnia* population (pre‐fish, high‐fish, and reduced‐fish) and thus evolution of a single natural *Daphnia* population as it occurred in nature, impacted chlorophyll *a* levels in the Control treatment, where the pre‐fish *Daphnia* mesocosms exhibited lower chlorophyll *a* concentrations than the reduced‐fish *Daphnia* mesocosms (cf. results of both linear mixed‐effect model and simplex projections; Figure 2 and Table 1A). Our experiment thus reveals differences in top‐down control of algae associated with the evolutionary response of a single *Daphnia* population as quantified over a period of a few years (pre‐fish to high‐fish: approximately 6 years; high‐fish to reduced‐fish: approximately 10 years). Yet, our results did not support our initial predictions that top‐down control in the absence of fish predation would be stronger in the pre‐fish population, while top‐down control in the presence of fish predation would be stronger in the high‐fish population. Instead, while top‐down control in the Control treatment decreased from the pre‐fish to the reduced‐fish population, we found no significant differences between populations in the extent of phytoplankton blooms in the presence of fish predation.

Both the linear mixed‐effect model and the simplex projections revealed differences in juvenile *Daphnia* dynamics between populations in the Control treatment, whereas in the Predation treatment, only the simplex projections revealed differences in *Daphnia* dynamics between populations. Our results indicate that evolution in this natural *Daphnia* populations did not only result in a differential top‐down control of phytoplankton but also in subtle differences in the dynamics of the *Daphnia* populations themselves.

Differences in dynamics often arise from differences in interactions between the state variables of the system (Chang, Ushio, & Hsieh, 2017; May, 1972; Mougi & Kondoh, 2012). In the following paragraphs, we discuss the differences in interactions between phytoplankton, *Daphnia* juveniles and *Daphnia* adults among populations and treatments that might explain the differences in top‐down control of algae by the different *Daphnia* subpopulations.

### 4.2 Interactions underlying the dynamics of juvenile and adult *Daphnia*

We observed striking differences among populations in the interactions estimated between juveniles, adults, and chlorophyll *a* in our time series analyses (e.g., Figure 4). S‐map estimates suggest that adult *Daphnia* in the Control treatment negatively affect juvenile abundances in the high‐fish and reduced‐fish populations but not in the pre‐fish population (Figure 3a). Adult *Daphnia* are the strongest competitors (De Roos et al., 1997; McCauley, Nelson, & Nisbet, 2008), and our results suggest that they decrease survival of the juveniles more in the high‐fish and reduced‐fish populations than in the pre‐fish population. This might reflect that populations adapted to fish predation pressure (here: high‐fish and reduced‐fish) in general produce more but smaller juveniles (Boersma et al., 1998; Riessen, 1999; Walsh & Post, 2011). Stoks et al. (2016) characterized the three populations for their life‐history traits, and juveniles of the pre‐fish population genotypes are indeed slightly larger than those of the high‐fish and reduced‐fish populations (see Supporting Information SI3, Fig. SI11 a).

In the Predation mesocosms, the S‐map estimates of interaction strength suggest that adult *Daphnia* have a negative impact on juveniles in all three populations. This is consistent with the fact that many studies have reported pronounced phenotypic plasticity in *Daphnia*, where animals exposed to fish kairomones often produce smaller offspring (Stibor & Lüning, 1994; Taylor & Gabriel, 1993). Admittedly, the data of Stoks et al. (2016) show divergent responses of neonate body length to the presence of fish kairomones in the different populations (Fig. SI11 a). Figure SI11 illustrates the relationship between average interaction strength of adults on juveniles (as estimated by S‐maps based on the time series in the different mesocosms) and three indices of juvenile quality: neonate size, 1/fecundity (assuming that the more juveniles a mother produces the less energy she can invest per individual juvenile), and size at maturity/fecundity (correcting for the fact that larger mothers might have more energy; see Supporting Information  SI3; all indices based on common garden life table data of Stoks et al. (2016)). These scatter plots are suggestive of a link between interaction strength and differences among populations in life‐history traits, putatively investment in individual juveniles and its associated starvation resistance (Gorbi, Moroni, Sandra, & Rossi, 2011).

The estimated impact of juveniles on adults is generally positive in all mesocosms, supporting the view that juveniles do not exert a competitive control on adults (Figure 3c,d). The effect of adults on adults differs among populations. In the Control treatment, the pre‐fish population shows a more positive effect of adults on adults than the other populations (Fig. SI4). The emerging picture on interactions between *Daphnia* is thus that (i) juveniles are competitively suppressed by adults, (ii) the extent to which this happens differs among populations as they evolved through time, (iii) juveniles do not competitively suppress adults but rather provide, through maturation, a source for new adults (De Roos et al., 1997; Gorbi et al., 2011; McCauley et al., 2008), and (iv) the effect of adults on adults is impacted by evolution, as adults of the pre‐fish populations have a stronger positive association with their own densities three days later than adults of the other populations in the Control treatment (Figure 5).

### 4.3 Interactions underlying the dynamics of chlorophyll *a*

Our S‐map estimates of the impact of *Daphnia* on phytoplankton indicate that the degree of top‐down control of algae is not mainly linked to the density of adults but to the density of juveniles. This estimated impact of juveniles is stronger at low ratios of adult *Daphnia* over phytoplankton biomass (Figure 4e,f). The presence of juveniles (rather than adults) has a high estimated impact on the top‐down control of algae when the densities of adults are low. While this link of dynamics in top‐down control to juvenile rather than adult *Daphnia* might at first sight be surprising, it needs to be viewed against the observation that throughout nearly the whole experiment in most mesocosms the *Daphnia* populations kept the phytoplankton at low densities. Top‐down control of phytoplankton by *Daphnia* is very strong during most of the experiment. As a result, the dynamics that are revealed by the S‐maps rather refer to the mechanisms through which the *Daphnia* populations temporarily lost their capacity to control phytoplankton blooms. We observed a temporarily higher phytoplankton biomass during only a limited time frame (from approx. day 40 till day 55 in most mesocosms). The dominance of strong top‐down control of algae during most of the experiment reflects the high grazing capacity of the large‐bodied water flea *D. magna*, which is well documented (Gianuca et al., 2016; Ye, Chang, García, Gong, & Hsieh, 2013). Given that grazing efficiency increases with body size (Brooks & Dodson, 1965; Mourelatos & Lacroix, 1990), this top‐down control is largely a function of the densities of adults, not juveniles. Yet, the S‐maps do not suggest this because efficient top‐down control of the algae is the dominant, almost invariable state in our experiments, precisely because large‐bodied *Daphnia* are such efficient grazers. Our analysis suggests a potential mechanism that leads to the occasional breakdown of this top‐down control, leading to a (temporary) algae bloom. The capacity of the *Daphnia* population to dynamically increase its grazing capacity depends on the presence of juveniles, which can grow and mature to replace dying and senescing adults. Our analysis reveals that through severe competition, the adult *Daphnia* suppress survival of the juveniles, and this results in a gradual decline in the juveniles to adult ratio during the period following the initial peak population density (see Figure 1). If there are no juveniles available that can grow into adults, any increase in growth rates in phytoplankton is translated into higher chlorophyll *a* levels, as the *Daphnia* population cannot increase its grazing pressure. This situation is temporary, because the higher food availability will then result in the adults producing offspring, with the resulting reproduction (McCauley, Murdoch, & Nisbet, 1990) reinstating the capacity of the *Daphnia* population to increase its grazing impact. This is what we observe: a rapid increase in phytoplankton biomasses that is, however, temporary, and algal biomasses become low again after the re‐appearance of juveniles in the populations.

Our analyses thus suggest a potential mechanism through which *D. magna* populations might (temporarily) lose the capacity to top‐down control phytoplankton biomass. This mechanism follows from the fact that adults suppress juveniles, while the latter are crucial to the capacity of the population to show an immediate numerical (in terms of number of adults) response to increasing food levels and thus represent the flexibility of the population to maintain a strong top‐down control on the algae under increasing nutrient loads. This proposed mechanism is directly related to the stage‐structured view on populations developed by De Roos, Schellekens, Van Kooten, and Persson (2008). It provides a link between the evolution of life‐history traits in response to changes in fish predation pressure in a natural population (Stoks et al., 2016) and changes in top‐down control of algae, through a differential negative impact of adult *Daphnia* on juveniles.

### 4.4 Methodological considerations

Our experiment suffered from some methodological problems and limitation. The first is related to the fact that we adjusted phosphorus levels at regular intervals in the Control but not in the Predation mesocosms. We aimed for a regular increase in nutrient concentrations in the mesocosms to prevent nutrient limitation and promote the growth of phytoplankton, thereby challenging the capacity of *Daphnia* population to achieve continued top‐down control of the phytoplankton. There was, however, no need to increase phosphorus levels in the Predation mesocosms as the presence of fish resulted in a spontaneous gradual enrichment. Every second time that we added P, we also added micronutrients. As a result of this procedure, micronutrients were added in the Control but not in the Predation mesocosms. This likely resulted in the higher phytoplankton biomasses in the Control compared to the Predation mesocosms. These higher phytoplankton biomasses in the absence compared to the presence of fish predation are opposite to expectations built on a rich literature on the impact of fish on algal blooms (Brönmark & Hansson, 2005; Scheffer, 1998). This difference in nutrient concentrations prohibits a direct comparison of phytoplankton concentrations of Predation and Control treatment, but does not interfere with comparisons of the dynamics of populations within treatments, and thus does not impact our interpretation of the results.

We note that, irrespective of the differences in nutrient concentrations, the low chlorophyll *a* levels in the mesocosms of the Predation treatment might also reflect the fact that the cages in which the fish were kept provided relatively good refuges for zooplankton. Even though the cages were quite large (1/3 of the volume of the mesocosms), they provided for a refuge of approximately 10–15 cm along the walls and bottom of the container. The efficiency of the refuge might have been enhanced by the fact that *D. magna,* when food stressed, engages in a browsing behavior, where they graze algae along hard surfaces (Horton, Rowan, Webster, & Peters, 1979). In doing so, they automatically were in a predator‐safe zone.

A second methodological problem is that our experiment suffered from contamination during inoculation of the mesocosms. More specifically, our high‐fish populations contained one clone from the reduced‐fish population and one clone from the pre‐fish population. Our reduced‐fish population contained one clone from the high‐fish population. The contaminants did not dominate the populations (see Supporting Information , Fig. SI1) and thus likely did not impact our results on differences in chlorophyll *a* concentrations and on S‐map estimated interactions between adults and juveniles and between these two life stages and chlorophyll *a*. If anything, this contamination made our observations on among‐population differences conservative.

We related our results to the life‐history data of Stoks et al. (2016, see also SI3). There are, however, some limitations associated with establishing this link. First, the number of data points was low (cf. three populations × two predation treatment conditions). Second, the life table data collected by Stoks et al. (2016) were assessed under optimal conditions of high food and low population densities, whereas the populations in our mesocosm experiment were exposed to widely varying population densities and food concentrations. These differences in context make it less straightforward to expect associations between the data in our mesocosm experiment (e.g., interaction strength) and the life‐history characteristics of the different populations. Yet, the tendencies revealed by the scatterplots linking interaction strengths of adults on juveniles with energy invested in newborns are suggestive.

## 5 CONCLUSION

Summarizing, our mesocosm experiment using resurrected *Daphnia* populations revealed a clear feedback of evolutionary trait change in a natural *D. magna* population that was exposed to changing levels of fish predation pressure on population dynamics and an ecosystem function, top‐down control of phytoplankton. Such a feedback of genetic differences in antipredator traits on top‐down control of algae was also observed by Walsh et al. (2012) in their study on *D. ambigua* populations from lakes with different levels of predation by alewife. Our results indicate that the differences in top‐down control that we observed in our experiment were a consequence of an evolutionary change resulting in the production of smaller juveniles, resulting in a stronger suppression of juveniles by adults in the evolved populations in the Control treatment. Our results suggest that the interactions between adults and juveniles can strongly impact the dynamics of populations and their top‐down effect on algae and can be modulated by rapid evolution.

The introduction of predators can cause complex dynamics with feedback loops that can cause alternative stable states (Scheffer, Carpenter, Foley, Folke, & Walker, 2001). In the context of shallow lakes, fish can eat large zooplankton, preventing them from suppressing the algae, which can lead to a regime shift to the turbid state (Scheffer, Hosper, Meijer, Moss, & Jeppesen, 1993). Our results, however, suggest that predators can also influence top‐down control of algae in more subtle ways, through the evolutionary responses they elicit when their densities are not so high as to entirely wipe out large zooplankton from the system. Our experimental results suggest that adaptive evolution in response to the presence of fish can facilitate a breakdown of top‐down control of algae through changes in demographic interactions. More specifically, the production of smaller offspring can lead to a stronger effect of adults on juveniles, which can lead to strong changes in population dynamics and consequences at the level of communities and ecosystems (De Roos & Persson, 2013). Top‐down control is a crucial ecosystem function in standing waters, and key to the ecosystem services ponds and lakes deliver to society (Moss, 2013; Scheffer, 1998). Algae blooms, and especially blooms to toxic cyanobacteria, strongly reduce the ecosystem services of ponds (e.g., aesthetic value, swimming water, production of drinking water, watering cattle) and might even cause health problems (Brooks et al., 2016).

Our study provides an example of an evolutionary response that fundamentally alters the processes structuring population dynamics and as a consequence also impacts ecosystem features. Our analysis is particularly strong because we could show these dynamics in a comparison of the behavior of resurrected populations derived from different time periods in the history of a single, natural population. Studies on ecosystem feedbacks of rapid evolution as it occurred in nature are a powerful new application of resurrection ecology.

## DATA ARCHIVING STATEMENT

Data available from the Dryad Digital Repository: https://doi.org/10.5061/dryad.7pc1h
