# Supplementary material for: Rapid evolution leads to differential population dynamics and top‐down control in resurrected Daphnia populations
Source: Evol Appl. 2017 Nov 15;11(1):96–111. doi: 10.1111/eva.12567 (PMC5748522; doi:10.1111/eva.12567)
Supplement: Supplementary file 1 [file EVA-11-96-s001.pdf]

Supporting information

SI1. *Genotype screening, clonal identities and contamination*

Genotype screening

The clonal identity of a total of 600 individuals, on average 25 randomly isolated individuals per mesocosm sampled at the end of the experiment (day 70), was determined by means of microsatellite analyses following Jansen, Geldof, De Meester, and Orsini (2011) and Orsini, Spanier, and De Meester (2012). Genomic DNA was extracted from random *Daphnia magna* individuals isolated from the mesocosms using the Proteinase K digestion method, as described by Mergeay et al. (2008). Live *Daphni*a were homogenized in 100 µl proteinase K-buffer (16mM $[NH_4]_2SO_4$, 67 mM Tris-HCl, pH 8.8, 0.01% Tween-20, 10% DTT and 0.5mM proteinase K). Following overnight incubation at 56°C, a 10 minute denaturation of samples was carried out at 96°C. Qualitative PCR (T1 PCR machine; Biometra, Germany) was conducted with the QIAGEN multiplex PCR kit (QIAGEN, Netherlands). Nine microsatellite markers, structured in one multiplex (multiplex MO1, as in Jansen et al. (2011) and Orsini et al. (2012); EST4276 was added as an additional marker) were used to identify clonal lineages.

PCR cycling conditions included an initial denaturation step at 95°C for 15 minutes, 30 cycles of 94°C for 30 seconds, annealing at 56°C for 30 seconds, extension at 72°C for 45 seconds, and a final elongation step at 60°C for 30 minutes. Microsatellite alleles were scored using an ABI PRISM 3031 automated sequencer (Applied Biosystems) and analyzed with the Gene Mapper software (applied Biosystems) using LIZZ500 as standard size. Based on reference samples (composed of animals from stock cultures and maternal individuals that gave rise to the animals that were used to stock mesocosms), all genotyped individuals were classified and named according to their layer of origin (B, M, T) and specific clone identification code (as in Pauwels, Stoks, Decaestecker, & De Meester, 2007; Stoks, Govaert, Pauwels, Jansen, & De Meester, 2016). The multilocus-genotypes were also compared to earlier genotyping efforts on the same clones (Orsini et al., 2012).

Clonal identities and contamination

The 36 clones used in the present experiment were the same as used in Stoks et al. (2016), however, we detected that some contamination had occurred when we screened the clones for their genotype using 9 microsatellite markers (see above). For the pre-fish population our multi-locus genotype identification suggests that one clone (B7) was accidently replaced by another clone from the same population (B9). For this population, the contamination only occurred within the population and resulted in 11 instead of 12 clones being used in the experiment (Figure SI1 a & b). For the high-fish population, two clones (M10 and M12) were accidently replaced by another clone from the same population (M11), one clone (M3) was accidently replaced by a clone with the same multilocus genotype as clone B6 of the pre-fish population, and one clone (M2) was accidently replaced by a clone with the same multilocus genotype as clone T12 from the reduced-fish population. So for this population, 8 clones were inoculated in addition to two clones that was derived from another population (Figure SI1 c & d). In the reduced-fish population, multi-locus genotype identification suggests that one clone (T3) was accidently replaced by a clone from the high-fish population (M7). For this population, 11 clones were used in the experiment in addition to one clone from another population (Figure SI1 e & f). As we screened the lineages for their genotypes after inoculating the mesocosms, we could not prevent the contamination. Although the contamination was unfortunate, only the among-population contaminations (one clone in the reduced-fish population and two clones in the high-fish population) can potentially interfere with the interpretation of our results, which are based on comparisons among populations. Moreover, to the extent that the interpretation of our results depends on the detection of among-population differences, the contamination does not induce false positives, but rather results in a conservative assessment of the impact of evolutionary change. The average detected relative abundance of the pre-fish clone in the high-fish population treatment at the end of the experiment was 0% in both Control and Predation treatments (Figure SI1

c & d), which translates into at most very low abundances of this clone. The average observed relative abundance of the reduced-fish population clone in the high-fish population treatment at the end of the experiment was 1% in the Control mesocosms and 26% in the Predation mesocosms (Figure SI1 c & d). The average observed relative abundance of the high-fish population clone in the reduced-fish population treatment at the end of the experiment was 38.1 % in the Control mesocosms and 28.7% in the Predation mesocosms (Figure SI1 e & f).

SI2. *Data analysis*

Empirical dynamic modeling

Empirical dynamic modeling (EDM), uses time-series to reconstruct the attractor manifold (see further) and allows for the exploration of the mechanisms underlying the dynamics of the system (Deyle, May, Munch, & Sugihara, 2016; Sugihara, 1994; Sugihara et al., 2012; Sugihara & May, 1990). Simplex projections are a forecasting technique from the EDM framework (Sugihara & May, 1990). The forecast skill of simplex projections using one group of time-series as a library (i.e. learning set) to make forecasts for data points in another time-series can be used to assess the similarity in the attractor manifold of those time-series. The S-map method is a technique from the EDM framework that can be used to estimate interaction strengths. S-maps do so by recovering the Jacobians (i.e. partial derivatives) at each time-point (Deyle, May, et al., 2016; Sugihara, 1994). Another technique from the EDM framework is convergent cross mapping (CCM) (Sugihara et al., 2012). CCMs can be used to identify causal links in the system. A brief explanation of EDM and the techniques that we used are given below, together with details of our implementation of them. For more in-depth explanations and further examples, we refer the reader to the rEDM user guide Ye, Clark, Deyele, Keyes, and Sugihara (2016) and empirical dynamic modeling for beginners (Chang, Ushio, & Hsieh, 2017).

Dynamic systems are often described as a set of multiple equations, in which each equation describes how the dynamics of a certain variable depends on itself and other variables. When

different states of a system are very similar, the state of the system will over the short run evolve very similarly. Representing all the relevant variables of a system as a set of Cartesian coordinates in state space and the observations in the time-series of these variables as coordinates visited by that system, results in a collection of trajectories forming a geometric object called an attractor manifold. The attractor manifold is the product of the specific rules and equations that describe the interactions between variables of the system, and thus is an empirical description of the dynamics of the system. An animation explaining the reconstruction of the attractor manifold by its variables is given in Sugihara et al. (2012; https://youtu.be/8DikuwwPWsY). As similar states evolve similarly over the short run, so do nearby states in state space. Thus, when time-series of the relevant variables are available, short-term forecasts for a given state can be made based on the predicted short-term future of nearby states in state space. However, when time-series for some variables in the system are not available, trajectories cross and nearby states will not go in exactly the same direction. In reality there might be countless variables influencing every system, but often the majority of the changes over time in a certain state variable are caused by only a few other variables. Hence, relatively skillful forecasts can be made based on these few relevant variables. It is, however, not always feasible to obtain measurements of or know all relevant variables of the system. Takens (1981) addressed this problem by using the fact that in a dynamic system, time-series of a variable that is influenced by other variables also contain information on these variables. An everyday used example of this principle is our ability to estimate the future location of moving objects by using consecutive snapshots of these objects, locations, without directly observing the momentum of the object and the forces (e.g. gravity) acting on it. Thus, instead of representing the state of the system as a vector (i.e. a multidimensional point) in state space with as coordinates the relevant state variables, one can use time-lagged observations (i.e. snapshots) of one variable as the coordinates. This is called a time-lagged embedding. There are a minimum number of time-lagged observations needed to capture all the necessary information and thereby prevent trajectories from crossing in the time-lagged embedding (i.e. for the embedding to be diffeomorphic). Before Takens' theorem, it

was not clear if the number of time-lags needed would be too high for any practical usage. Takens (1981) found a connection between the number of required lags, i.e. the embedding dimension *E*, and the number of state variables, i.e. the number of dimensions *D*. He demonstrated that a time-lagged embedding using just $E = 2 * D + 1$ lags is the maximum needed to obtain a diffeomorphism of the original attractor manifold of a dynamic system (i.e. to prevent lines crossing in the embedding). Thus, if the relevant variables of a system are the two variables X and Y, no more than five lags are needed in the embedding (i.e. {Y(t), Y(t-1), Y(t-2), Y(t-3), Y(t-4)}). This means one can obtain a shadow version (i.e. a time-lagged embedding) of the original attractor manifold by using only a few lags of one variable. Although this shadow manifold is a globally distorted (e.g. stretched or bent) version of the original manifold, this distortion is a smooth invertible change in coordinates. A short animation by Sugihara et al. (2012) explaining Takens' theorem can be found here: https://youtu.be/QQwtrWBwxQg. The same points in time that are close on the original attractor manifold are also close on the shadow manifold. Thus, as the state of the system changes over time and visits different neighborhoods on the attractor manifold, it will pass by neighborhoods on the manifold that it has visited before and the time points in history close to each other on the original attractor are also close on the shadow manifold. In (univariate) simplex projections, this fact is used to make forecasts using only one variable (Sugihara & May, 1990). As differences in dynamics between different populations can be more pronounced in some variables (i.e. dimensions in state space) than others, we decided to test for significant differences between populations independently for different variables. For this test we were thus able to use univariate simplex projections.

An interesting consequence from Takens' theorem is that when a variable *X* influences another variable *Y*, then *X* will leave its mark on the dynamics of *Y*. Time-points close on the time-lagged embedding of *X* will also be close on the time-lagged embedding of *Y*. Thus, if nearby points on the shadow manifold of *Y* are also close on the shadow manifold of *X*, than *X* likely influences variable *Y*. This is the basis for convergent cross mapping (Sugihara et al., 2012). An animation explaining this principle by Sugihara et al. (2012) can be found here: https://youtu.be/NrFdIz-D2yM.

Simplex projections

We used univariate simplex projections to compare the similarity in dynamics of individual variables between populations. In simplex projections, for each state in the time-lagged embedding of the prediction set (i.e. for each target state $\underline{x}(t^*)$), the $E+1$ closest points on the shadow manifold of X, which is reconstructed using only the library set, are taken and a weight is calculated based on their Euclidian distance from $x(t^*)$. To make forecasts with a specified time step, t_p, into the future, the states that the $E+1$ nearest states have t_p into the future are multiplied with their respective weights and the average of these products is used as the forecast(Sugihara & May, 1990). If the time-series used in the library can be used to make skillful forecasts of the time-series in the prediction set, then the dynamics underlying the time-series in the prediction set must be similar to those in the time-series used in the library. We expressed forecast skill in MAE (mean absolute error). For each population, to determine whether the dynamics of replicates of the same population are more similar than between different populations, we used a one sided Mann-Whitney test comparing the skill of within population forecasts to forecasts from other populations. We made forecasts for all possible combinations of (replicate) time-series as library and prediction sets, with 3 time-series as library predicting one other time-series (i.e. excluding combinations where the same time-series occurred in both library and prediction set). This leads to four MAEs for each set of within population forecasts and 16 for the between population forecasts. For simplex projections the number of lags used in the reconstructed state space has to be specified (i.e. the embedding dimension E). We here each time tried all embedding dimensions below 7 and used the E that resulted in the highest value.

Another parameter that has to be set in simplex predictions is the forecast time step t_p. The standard choice for this is to use the smallest step (i.e. 3 days for *Daphnia* time-series and 1 day for phytoplankton). However, when dynamics are relatively slow, small time steps might not be sufficiently challenging to distinguish in forecast skill. In contrast, as is characteristic to non-linear systems, large forecast time steps result in lower predictability and forecast skill decreases in all models. Therefore, in testing the difference between populations, we tested different forecast time-

steps (3, 6, 9, 12 and 15 days). Although within population simplex projections might not be significantly more skillful (i.e. lower MAE) with very small time-steps or too large time-steps, if they significantly differ from another population, than at least within a certain range of forecast time steps, they would do significantly better when using libraries from the same population than when using libraries from a different population. In Simplex projections, unlike the other EDM methods we applied, each time-series is analyzed separately. Hence, we were able to make use of the finer resolution of the phytoplankton time-series and use a time lag of one day between the time lags in the embedding. The P-values of these tests against the forecast time-step used are shown in figure SI8 and the number of significant p-values (<0.05) among the tests using the five different time steps, are shown in Table SI1 in the main text.

Cross mapping

For each time point t_p, the *E+1* closest points on the shadow manifold of *Y* are taken and a weight is calculated based on their distance from t_p. Multiplying the *E+1* nearest states with their respective weights and averaging them gives the value of *Y* at t_p. The same *E+1* time points, but from the time-series of *X* are then multiplied with these weights to make a prediction. Note that these do not have to be the *E+1* closest points on *X* as well, as long as they are close enough, the prediction will be reasonably skillful. The Pearson correlation coefficient between the predicted values of *X* based on the manifold of *Y* at each time point and the true value of *X* at those time points is the prediction skill of the cross map (ρ_{ccm}). In the presence of a causal link between the considered variables, the prediction skill will increase until they converge to a certain ρ_{ccm} when more time-points are used to make the shadow manifold of the library variable (in our case *Y*), (Sugihara et al., 2012).

Given that each selection of days from the time-series to use in the library will result in a different cross map skill (ρ_{ccm}), the days of the time-series used at each library size is drawn multiple times (in our case 100 times) resulting in a distribution of cross map skills (ρ_{ccm}). In all our analysis, we combined time-series data of the four replicates of each population within each treatment, but in

such a way that no one vector (i.e. set of time lagged observations) contained data points from different replicates (Clark et al., 2015; Hsieh, Anderson, & Sugihara, 2007). To avoid problems with overfitting, we first performed CCMs predicting the state of *X* three days before based, on the shadow manifold of *Y* (see Deyle, Maher, Hernandez, Basu, & Sugihara, 2016; Deyle, May, et al., 2016) and then used the embedding dimension resulting in the highest ρ_{ccm} from this as embedding dimension for the actual CCMs. In the actual CCMs, the shadow manifold of Y was used to predict the state of X on the same day (see Figure SI10). False signs of positive cross maps were further eliminated by testing against randomly generated surrogate time-series that were used as a null distribution (Deyle, Maher, et al., 2016; Deyle, May, et al., 2016). We generated 500 surrogate time-series in which the order of the days was randomly permutated. If the cross map skill is significantly greater in the original time-series than in the surrogate time-series based null distribution, the properties that were incorporated in the surrogate time-series are not enough to explain the size of the cross map skill. Importantly, we used the same permutation of days for each of the 4 replicates in generating null distributions to also consider potential false signs of causal influences resulting from synchrony of variables with external forces that acted upon the four replicates simultaneously. We determined the embedding dimensions for both the surrogate and the regular time-series similarly to the regular CCMs (i.e. based on the ρ_{ccm} in 3 day backward predictions). When the original time-series performed better than 95% of the surrogates using ρ_{ccm} as a criterion, we considered them to be significant (i.e. $p<0.05$). Results of this analysis are given in Figure SI9. All cases with significant surrogate tests (Figure SI9) showed convergence in the CCM plots (Figure SI10). An overview of significant interactions between variables is given in Figure 5 in the main text.

S-maps

The closer one zooms into a small neighborhood on the attractor manifold, the more linear the manifold becomes. The S-map method is a locally weighted linear regression scheme (Sugihara, 1994). It approximates the best local linear model at each measured state by giving more weight to

states on the attractor manifold that are more nearby that state. Similar to a multivariate linear regression, S-maps average out noise by using all data points, rather than just a few neighboring points in state space. In contrast to multivariate linear regression, S-maps allow points more closely located on the manifold to the target point $\underline{x}(t)$ to be given a higher weight in the forecast, thereby accounting for potential state-dependent-differences in interaction strengths over time, which is typical for non-linear dynamic systems. S-maps contain one variable, theta (θ), which sets the degree of non-linearity by determining to what extent points more nearby on the attractor manifold are given more weight than distant ones. A theta of zero leads to equal weights for all points and basically results in a simple multivariate linear regression. As each target point $\underline{x}(t)$ is positioned differently on the manifold with respect to the other points in the dataset, a separate weighted linear regression is made for each location $\underline{x}(t^*)$ on the manifold. The weight given to observation *i* when making the local linear approximation of $\underline{x}(t^*)$ is given by $w_i = e^{\frac{-\theta\|\underline{x}(t_i)-\underline{x}(t^*)\|}{\bar{d}}}$, where $\|\underline{x}(t_i) - \underline{x}(t^*)\|$ is the Euclidian distance between the two vectors and $\bar{d} = \frac{1}{n}\sum_{i=1}^{n}\|\underline{x}(t_i) - \underline{x}(t^*)\|$. With the separate local weightings around each target point, separate linear regressions can be made by solving the SVD (singular value decomposition) for $\boldsymbol{C}$ in the linear equation $\boldsymbol{B} = \boldsymbol{A} \cdot \boldsymbol{C}$, where **A** is the $n{\times}E$ dimensional matrix (*E* is the embedding dimension or number of variables used to reconstruct the attractor manifold) of states $\underline{x}_j(t_i)$ weighted based on the proximity to the target states $\underline{x}(t^*)$ given by $A_{ij} = w_i x_j(t_i)$, ***B*** is the *n*-element vector of future values of the target variables x_k, also weighted based on the proximity to the target states, given by $B_i = w_i x_k(t_i + 1)$, and ***C*** is the *E*-element vector of Jacobian elements at the target point $\underline{x}(t^*)$ for the target variable x_k.

S-maps can be used on both univariate embeddings of one variable or using a multivariate embedding (Deyle & Sugihara, 2011). When using a multivariate embedding, the S-map coefficients, i.e. the regression coefficients of each locally weighted linear regression (which are equivalent to the partial derivatives on the manifold or the Jacobian) give the strengths and signs of the interactions between variables (Deyle, May, et al., 2016).

In our study, all the forecasts were done 3 days into the future, so that direct comparisons with our observed data could be carried out, and using leave-one-out cross validation. We made separate library sets for each of the 3 populations in each treatment (6 library sets in total) and always used the same multivariate embedding: {Chla(t), Adults(t), Juv(t)}, irrespective of which of the three target variables (x_k) (Chla, Juveniles or Adults) was used. We performed S-maps with the following theta values: 0, 10^{-4}, $3*10^{-4}$, 0.001, 0.003, 0.01, 0.03, 0.1, 0.3, 0.5, 0.75, 1, 1.5, 2, 3, 4, 6, 8, 10, 15 and 20. The theta resulting in the best forecast skill ρ was used for each population, treatment and forecasted variable. The forecast skill ρ was found to be always significantly (<0.0001) better than zero using Fisher's z-transformation (Table SI2). The resulting S-map estimates of interaction strengths (i.e. the elements of ***C***) are plotted against time in Figure SI6 and SI7.

The effect of *X* on the future (3 days later) of *Y* is given by $\frac{\partial X(t+3)}{\partial Y(t)}$. The interaction strengths are calculated separately at each time point in the time-series. At each time point, based on the location of the state in state space, a locally weighted linear regression is performed. For Adult *Daphnia*, for instance, a linear regression would look like this:

$$Adult(t+3) = \beta_0 + \beta_1 Chla(t) + \beta_2 Adult(t) + \beta_3 Juv(t) + \epsilon_t.$$

But in the non-linear forecasts by the S-maps, for each forecast different parameters are estimated because of the local weighting, and the β_1, β_2 and β_3 become the elements of the Jacobian matrix:

$$\beta_1 = \frac{\partial Adult(t+3)}{\partial Chla(t)}, \beta_2 = \frac{\partial Adult(t+3)}{\partial Adult(t)}, \beta_3 = \frac{\partial Adult(t+3)}{\partial Juv(t)}.$$

Non-linear systems, as analyzed by S-maps, can thus be interpreted as linear systems, but with changing parameters depending on the position in state space of the system. By interpreting interactions this way, the straightforward intuition we have with linear systems can be extended to explore non-linear systems, the difference being that the interaction strengths change over time depending on the states. So we see a cloud of interaction strengths in Figure 3, Figure 4, Figure SI4 and Figure SI5 that shows how interaction strengths vary with state. By plotting the interaction

strengths estimated at each time point against measured observations at the same time points of an important variable (e.g. phytoplankton in Figure 3, Figure 4a-d, Figure SI4 and Figure SI5) we are able to see how the interaction strengths from the locally weighted linear regressions predicted by the model depend on this variable. To test for the significance of relations between estimated interaction strengths and variables, we performed several linear regressions (Figure 4). The statistics of these linear regressions are shown in Table SI2.

SI3. *Linking interaction strengths to genotypic trait values*

Figure SI11 contains scatter plots showing the association between average interaction strengths of adults on juveniles against three measures of juvenile 'quality' based on the life history data provided by Stoks et al. (2016): (A) average neonate size (body length); (B) inverse average number of offspring of the first two clutches (1/average fecundity); (C) average size at maturity divided by average fecundity. All data points represent averages of all clones of a given population and in either control or fish kairomone exposure conditions as studied in the common garden experiment carried out by Stoks et al. (2016).

All three variables capture some aspects of juvenile energy content, but should be considered loose approximations rather than precise estimates. We plot neonate body size because of its link to body mass. We plot average number of offspring assuming that investment per individual offspring would become lower as their numbers increase. This assumes an equal amount of available energy of all mothers. The third index (average size at maturity divided by average fecundity) tries to take variation in energy content of mothers into consideration by scaling the number of offspring to the size of the mother.

All three scatterplots are suggestive for a relationship between the interaction strengths of adults and juvenile 'quality', even though none of the relationships is significant. The lack of significance might reflect: (i) the limited number of data points, (ii) the approximate nature of our measures of energy content, (iii) the fact that the life history data are limited to size of neonates, size of adults,

and number of offspring, whereas the mesocosm data integrate information on different age classes within the categories of juveniles and adults, and (iv) the impact of density effects on body sizes (all life history data in Stoks et al. (2016) were collected under optimal food conditions). Given that Stoks et al. (2016) quantified among population differences in genotypic trait values under highly standardized conditions, while the mesocosm experiment quantified dynamics of these same populations under highly fluctuating population densities and food conditions, we consider the observed relationships (with correlation coefficients reaching 0.59) to be suggestive of a mechanistic link between the life histories of the populations and the observed dynamics of *Daphnia* and chlorophyll a in the mesocosms.

Table **SI1**. Degree of non-linearity (theta) resulting in optimal S-map forecast skill (i.e. highest ρ) and probability that the ρ is greater than zero using Fisher's z-transformation.

Treatment	Population	Forecasted variable:								
		Chlorophyll a			Adults			Juveniles		
		theta	ρ	probability (Fisher)	theta	ρ	probability (Fisher)	theta	ρ	probability (Fisher)
Control	Pre-fish	1	0.65	1.89E-10	0.75	0.59	2.38E-08	0.1	0.68	1.21E-11
	High-fish	1.5	0.57	1.18E-07	0	0.52	1.42E-06	0.5	0.55	2.63E-07
	Reduced-fish	2	0.69	3.29E-12	0	0.79	2.60E-18	0	0.70	1.06E-12
Predation	Pre-fish	1	0.59	2.87E-08	6	0.79	2.75E-18	1	0.68	1.32E-11
	High-fish	2	0.68	1.23E-11	3	0.84	4.45E-23	1.5	0.77	1.52E-16
	Reduced-fish	4	0.53	1.27E-06	0.5	0.74	9.27E-15	0.75	0.68	1.06E-11

Table **SI2**. Results of the simple linear regressions in Figure 4. The S-map estimates of the effect of *Daphnia* on phytoplankton were regressed on chlorophyll *a* concentration (log(chla)) and (only for juveniles) the ratio of Adult *Daphnia* : chlorophyll *a* (Adult/log(chla)) for pre-fish, high-fish and reduced-fish in the Control and Predation treatments. Linear regressions were only performed for all population x treatment combinations when the interactions were significant ($p<0.05$) in the CCM test.

		Pre-fish				High-fish				Reduced-fish			
Treatment	Regression	R^2	R^2adj	$F_{1,62}$	p	R^2	R^2adj	$F_{1,62}$	p	R^2	R^2adj	$F_{1,62}$	p
Control	∂Chla/∂Adult on log(Chla)	ρCCM is not significant				ρCCM is not significant				ρCCM is not significant			
	∂Chla/∂Juv on log(Chla)	0.68	0.6776	133.40	< 0.001	ρCCM is not significant				0.49	0.4804	59.25	0.001
	∂Chla/∂Juv on Adult/log(chla)	0.33	0.3239	31.19	< 0.001	ρCCM is not significant				0.12	0.1038	8.298	< 0.01
Predation	∂Chla/∂Adult on log(Chla)	0.45	0.4411	50.73	0.001	0.25	0.2421	21.13	< 0.001	ρCCM is not significant			
	∂Chla/∂Juv on log(Chla)	0.09	0.078	6.30	< 0.05	0.39	0.3767	39.08	< 0.001	ρCCM is not significant			
	∂Chla/∂Juv on Adult/log(chla)	0.22	0.2084	17.58	< 0.001	0.47	0.4621	55.12	< 0.001	ρCCM is not significant			

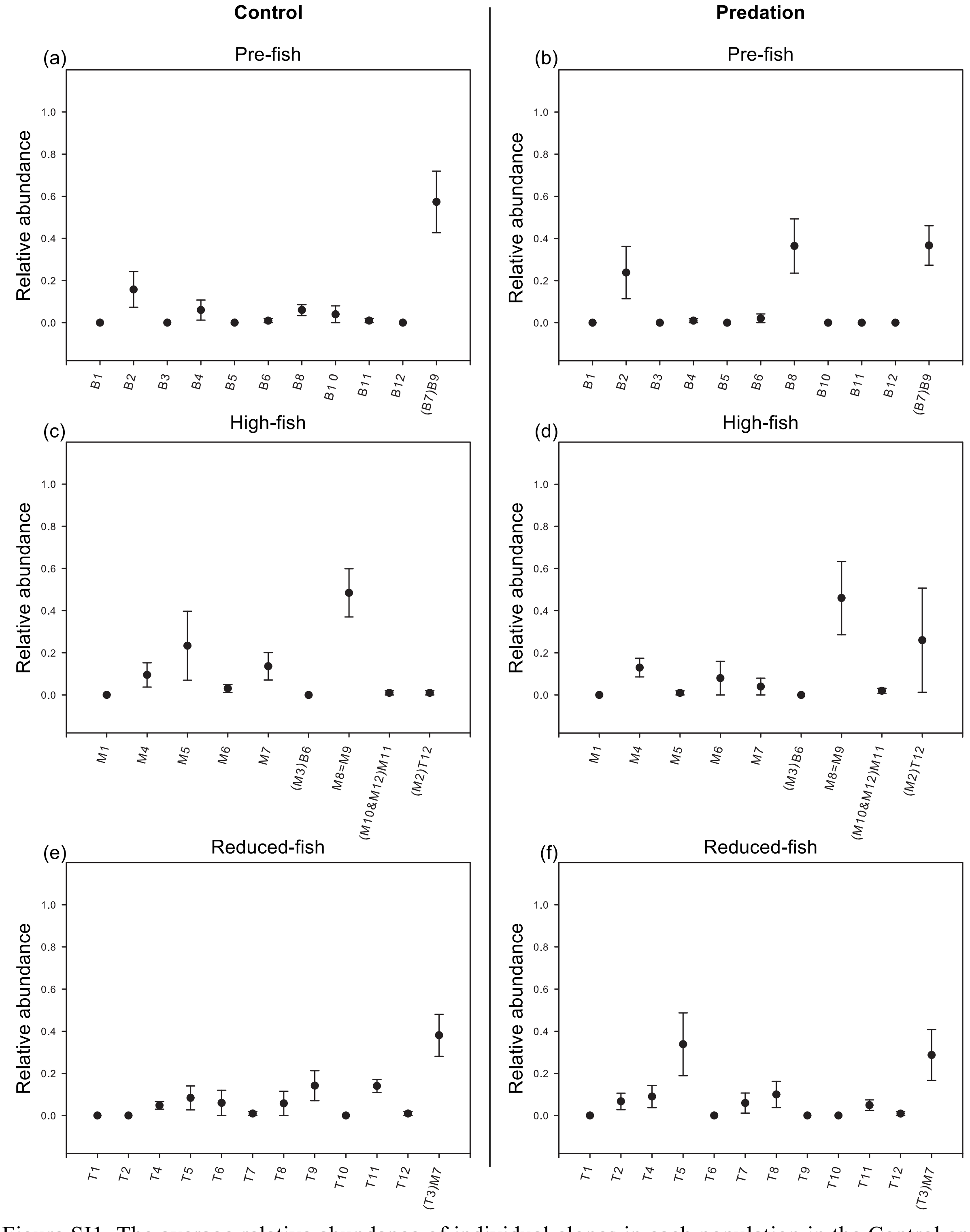

Figure SI1. The average relative abundance of individual clones in each population in the Control and Predation treatment at the end of the experiment. Clones codes that start with a letter "T" belong to the reduced-fish population ("Top"), codes starting with "M" indicate clones that belong to the high-fish population ("Middle") and "B" indicates clones from the pre-fish population ("Bottom"). In the pre-fish population clone B7 was accidently replaced by clone B9 from the same population. In the high-

fish population clones M8 and M9 are the same multi-locus genotype (cannot be discriminated with the marker set used), clones M10 and M12 were accidently replaced by clone M11 from the same population, clone M3 was accidently replaced by a clone with the same multilocus genotype as B6 of the pre-fish population and clone M2 was accidently replaced by a clone with the same multilocus genotype as clone T12 from the reduced-fish population. In the reduced-fish population clone T3 was replaced by clone M7 from the high-fish population. Error bars indicate standard error.

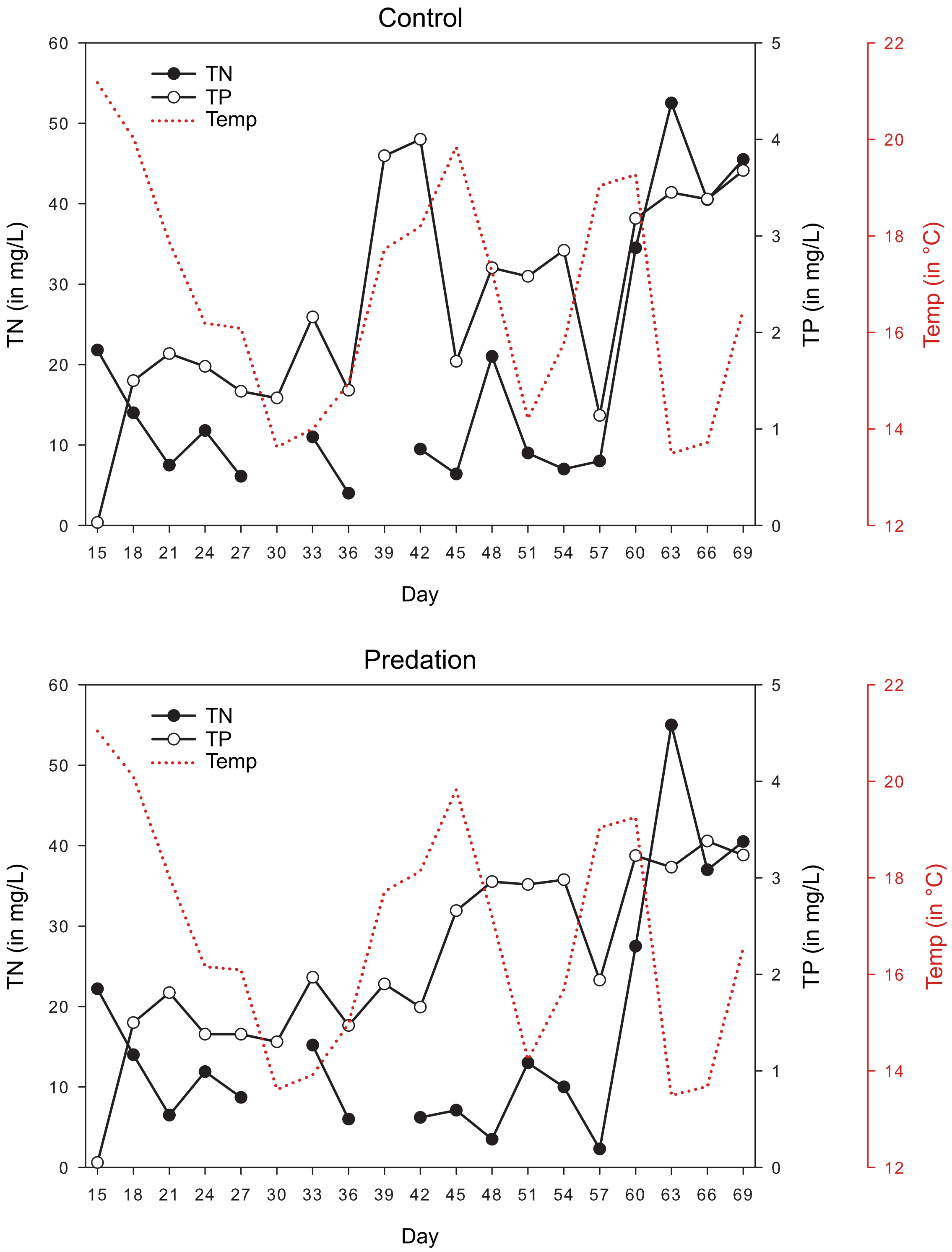

Figure SI2. Total nitrogen concentration (TN), total phosphorus concentration (TP) and water temperature (Temp) for each time point over the duration of the experiment based on pooled samples of the Control (upper panel) and Predation (lower panel) treatment mesocosms separately .

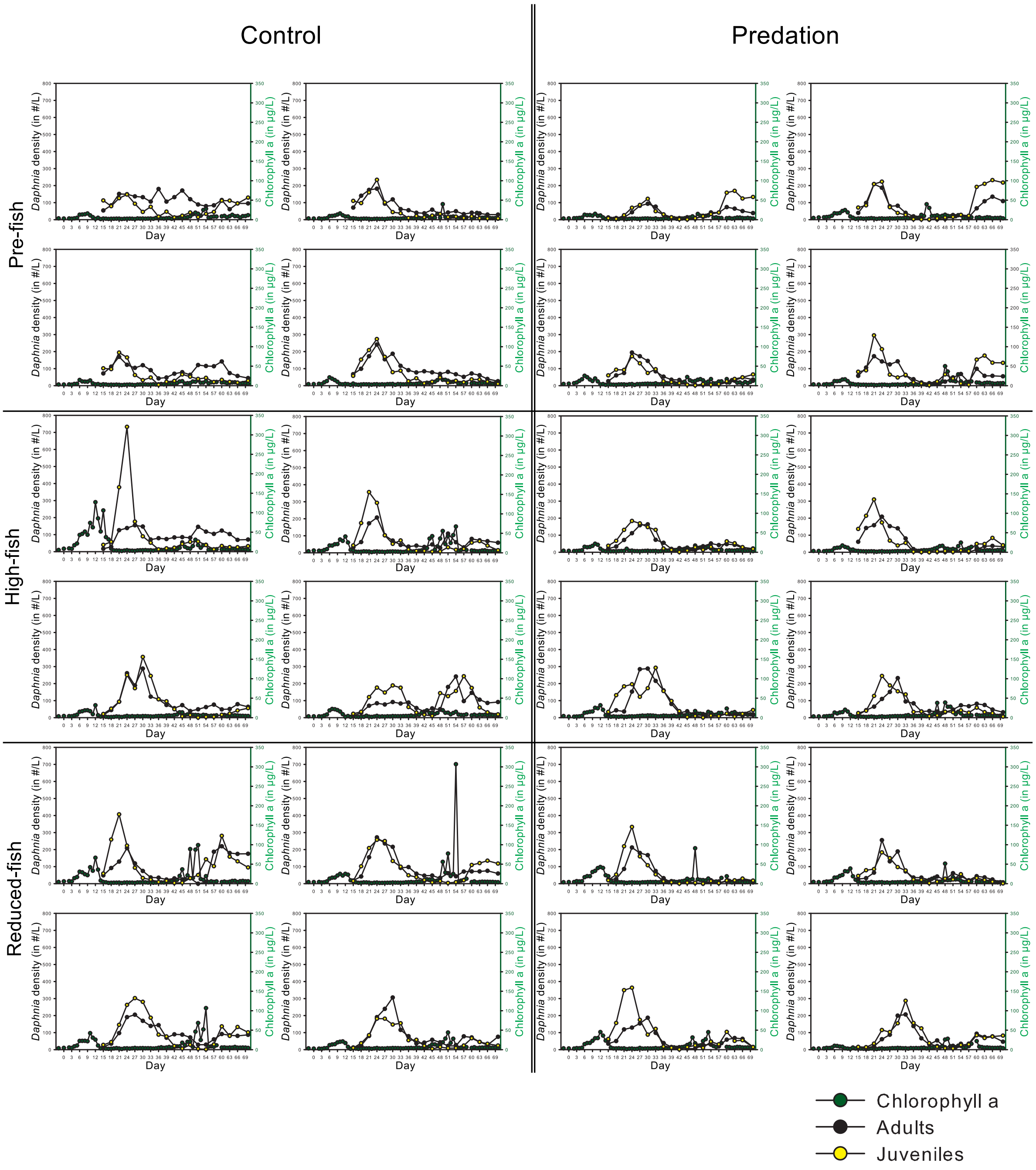

Figure SI3. The density of *Daphnia* adults, *Daphnia* juveniles, and chlorophyll *a* concentration at each time point for each replicate mesocosms of each population separately in the Control and Predation treatments.

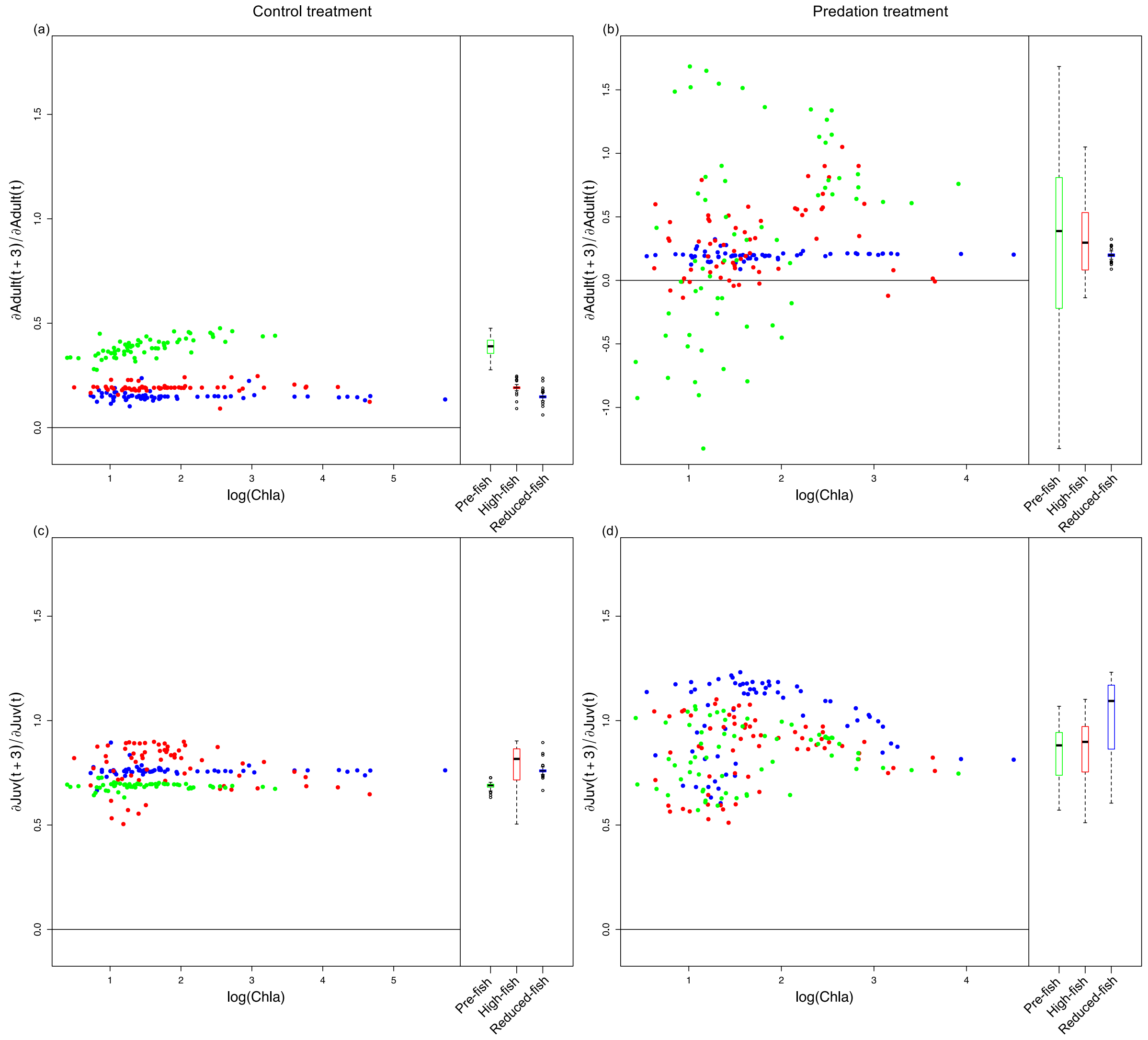

Figure SI4. The effect of adults on adults [∂Adult(t+3)/∂Adult(t)] (a,b) and the effect of juveniles on juveniles [∂Juv(t+3)/∂Juv(t)] (c,d) as a function of the phytoplankton biomass (log(Chla) for the three populations: • pre-fish, • high-fish and • reduced-fish, in the Control (a,c) and Predation (b,d) treatments. The effect of adults and juveniles on their own future densities is a mixture of negative effects from resource competition with themselves and positive effects from survival. Boxplots show the distribution of estimated interaction strengths for the three populations. The bottom and top of the box show the lower and upper quartiles, the band in between them shows the median, whiskers show the minimum and maximum (excluding outliers) and circles show the outliers. Outliers are values more than 1.5 times the length of interquartile range greater than the upper quartile or smaller than the lower quartile. The S-map estimated interaction strengths are in normalized units. The solid line shows the line of no effect.

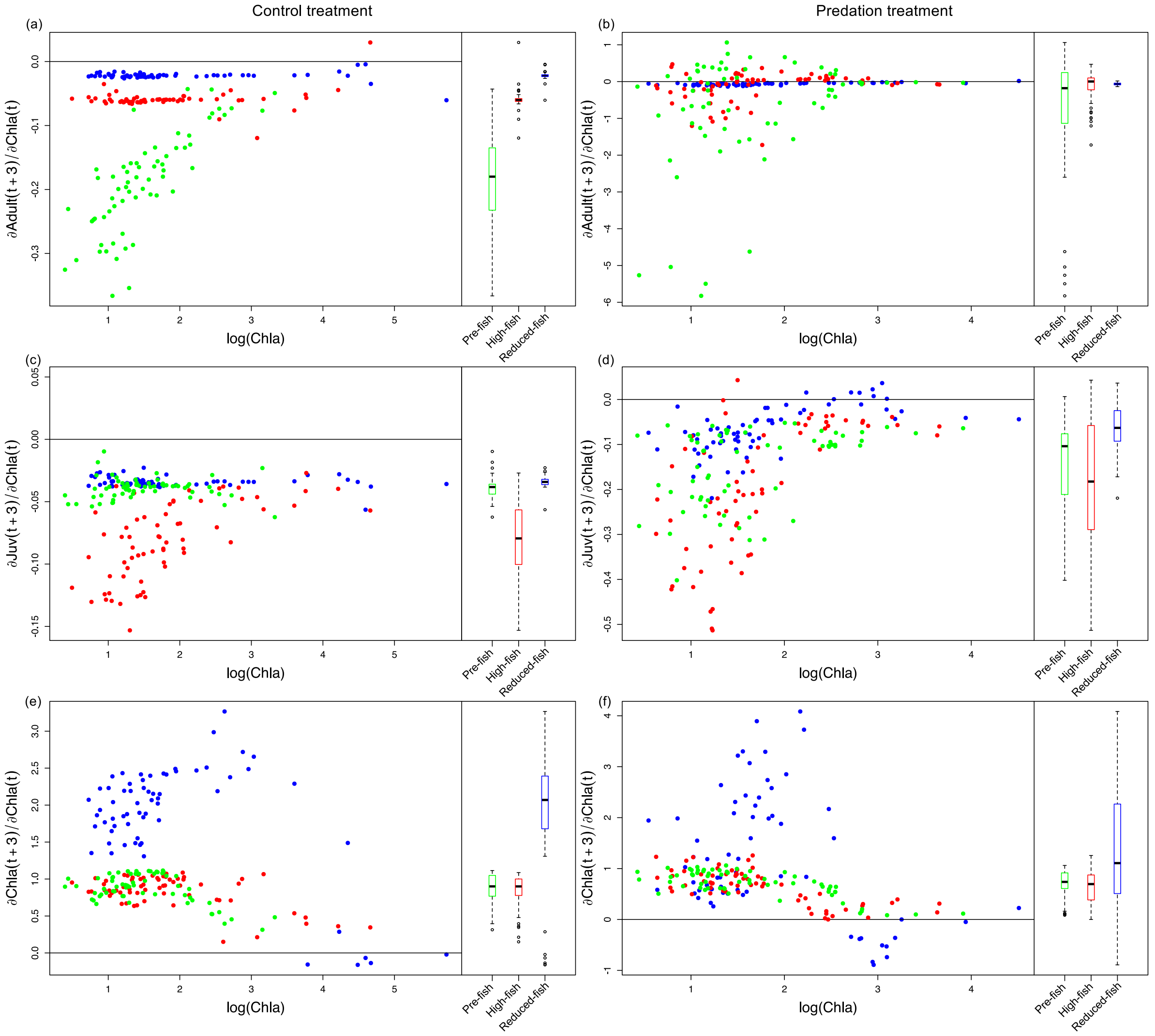

Figure SI5. The effect of phytoplankton on *Daphnia* adults [∂Adults(t+3)/∂ Chla(t)] (a,b) on *Daphnia* juveniles [∂Juv(t+3)/∂ Chla(t)] (c,d), and on phytoplankton itself [∂Chla(t+3)/∂ Chla(t)] (e,f) as a function of the phytoplankton biomass (log(Chla)) in the three populations: • pre-fish, • high-fish and • reduced-fish, in the Control (a,c,e) and Predation (b,d,f) treatments. Boxplots show the distribution of estimated interaction strengths for the three populations. The bottom and top of the box show the lower and upper quartiles, the band in between them shows the median, whiskers show the minimum and maximum (excluding outliers) and circles show the outliers. Outliers are values more than 1.5 times the length of interquartile range greater than the upper quartile or smaller than the lower quartile. The S-map estimated interaction strengths are in normalized units. The solid line shows the line of no effect.

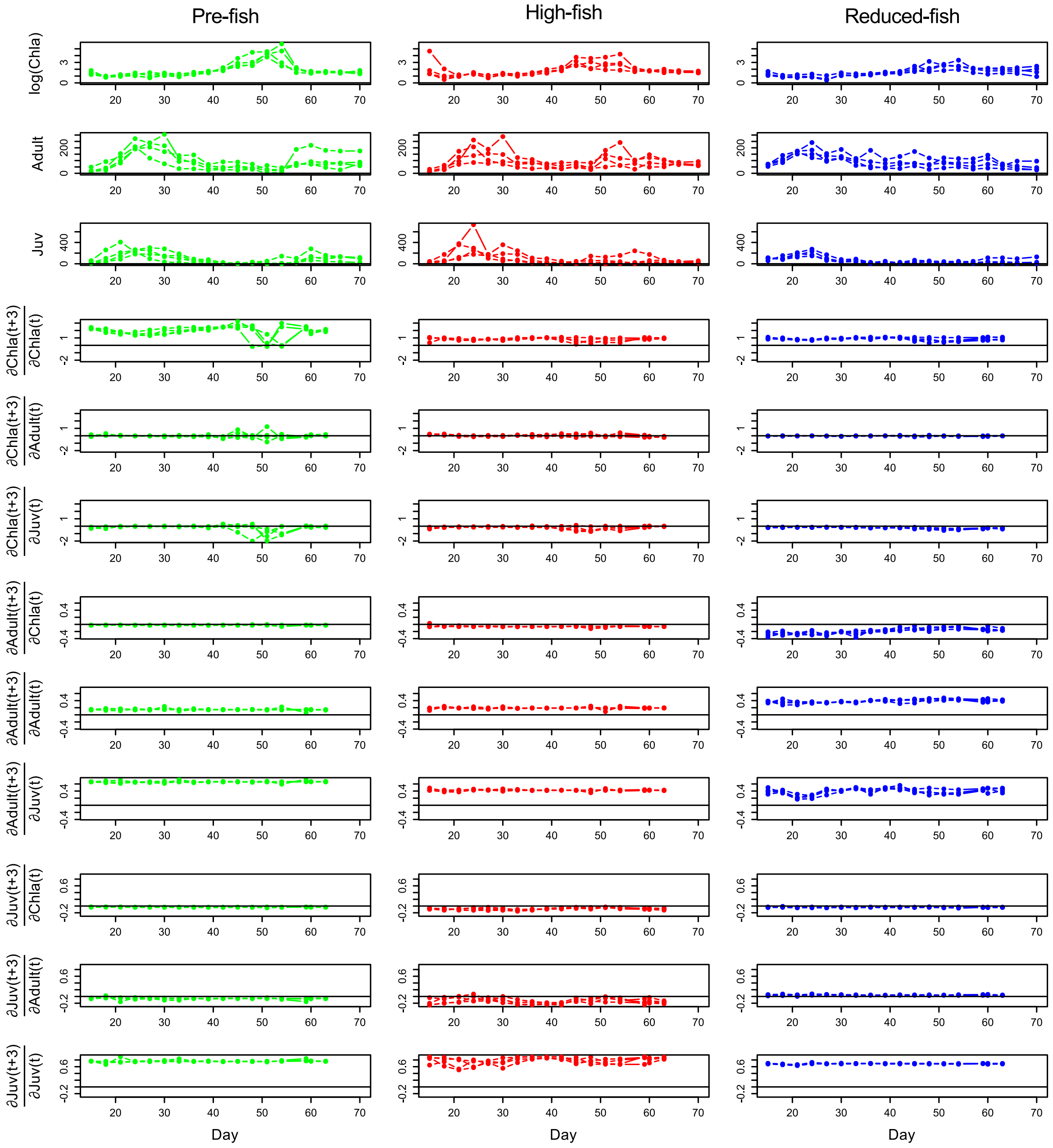

Figure SI6. Dynamic interactions in the Control treatment. The top three rows show the time-series of the three variables (phytoplankton given by log(Chla) (in log(µg/L)), adult densities (in #/L) and juvenile densities (in #/L)); the other rows show all the different S-map estimated interaction strengths as a function of the day. Row 4-6 show effects on phytoplankton, row 7-9 show effects on adults and row 10-12 show effects on juveniles. The first column contains the • pre-fish population, the second the • high-fish population and the third the • reduced-fish population. The S-map estimated interaction strengths are in normalized units. The solid line shows the line of no effect.

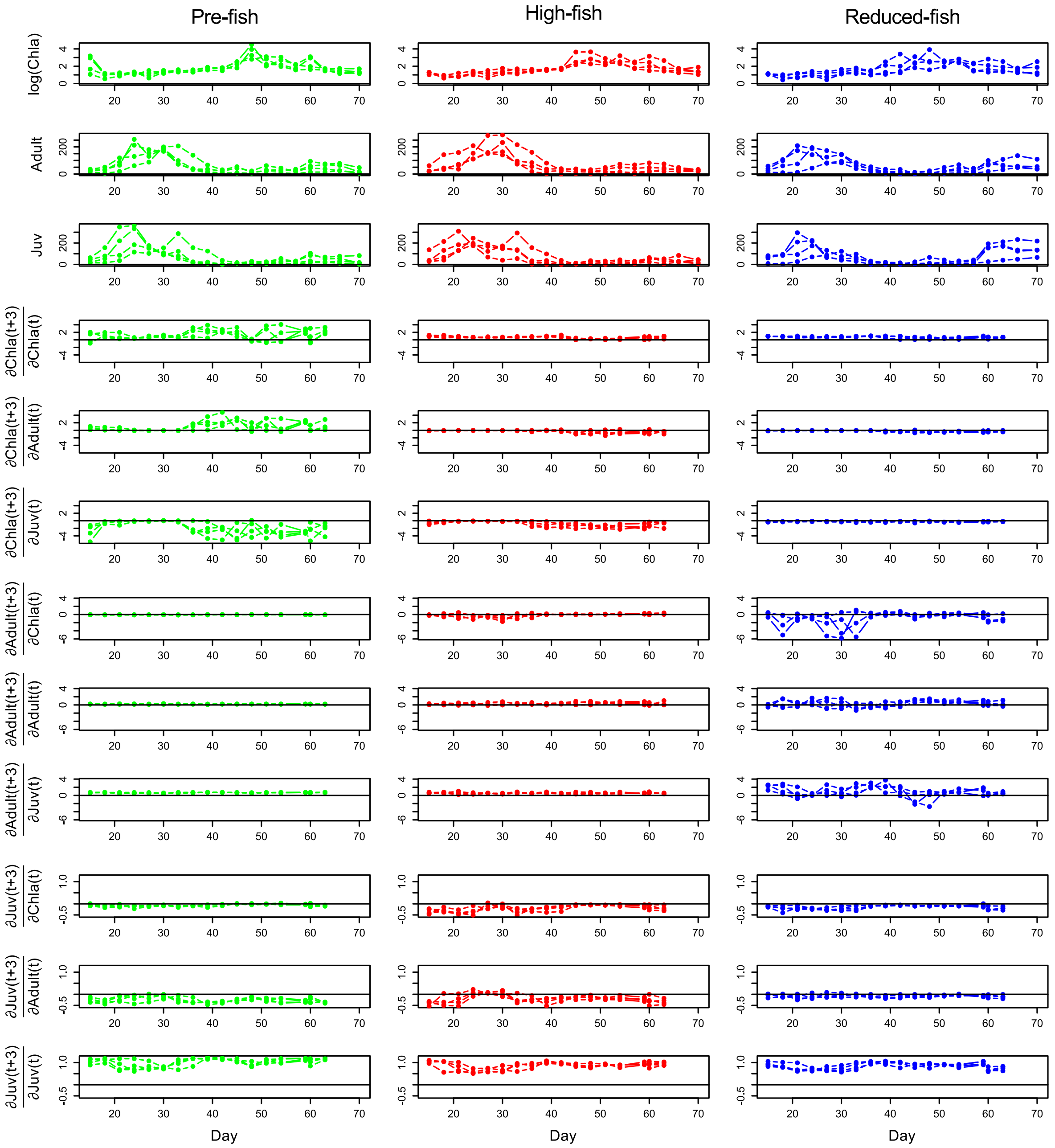

Figure SI7. Dynamic interactions in the Predation treatment. The top three rows show the time-series of the three variables (phytoplankton given by log(Chla) (in log(μg/L)), adult densities (in #/L) and juvenile densities (in #/L)); the other rows show all the different S-map estimated interaction strengths as a function of the day. Row 4-6 show effects on phytoplankton, row 7-9 show effects on adults and row 10-12 show effects on juveniles. The first column contains the • pre-fish population, the second the • high-fish population and the third the • reduced-fish population. The S-map estimated interaction strengths are in normalized units. The solid line shows the line of no effect.

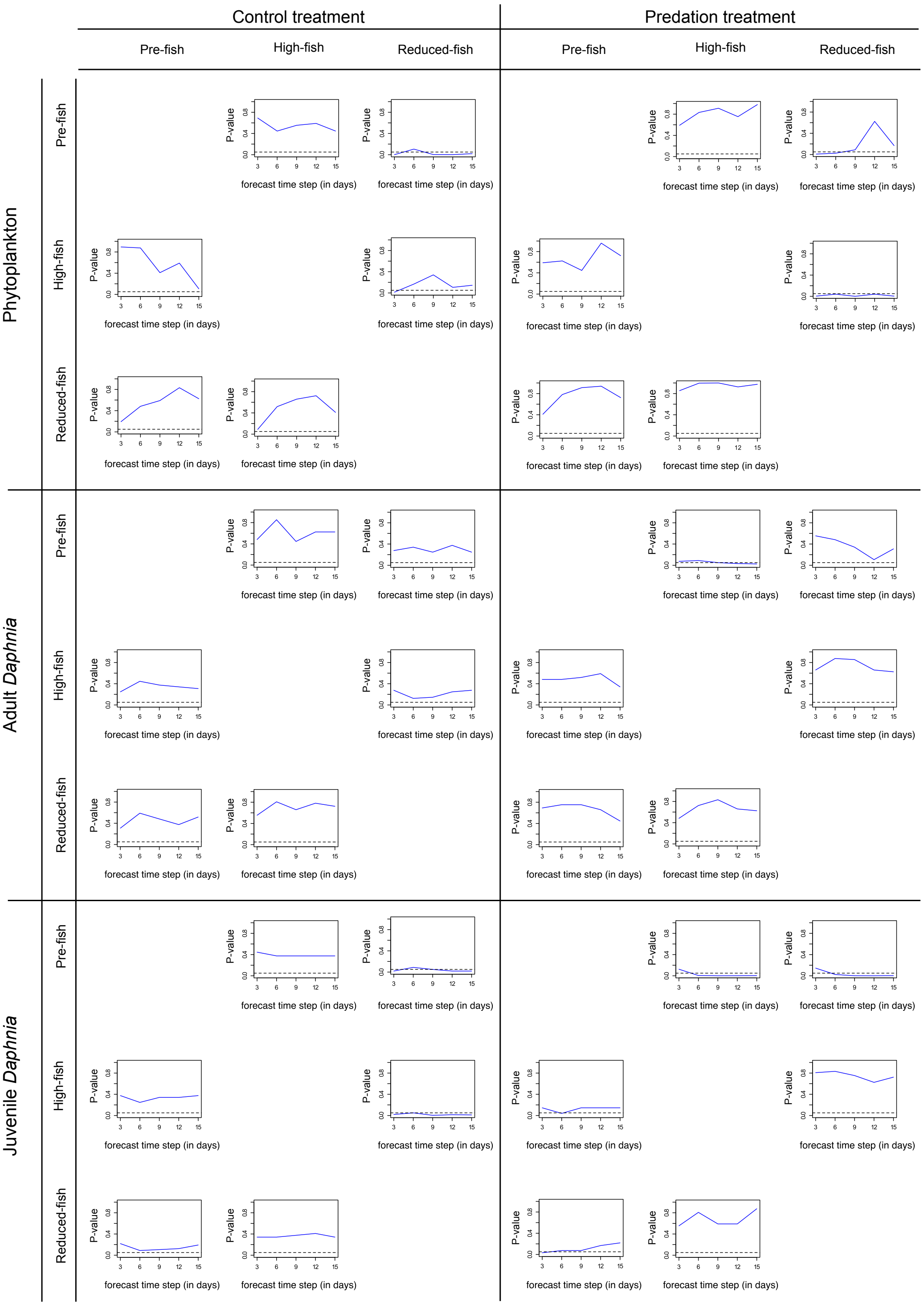

Figure SI8. Simplex projection tests for population differences. One sided Mann-Whitney tests were used to determine if forecasts between replicate mesocosms of the same population are more skillful than predictions from a replicate mesocosms from another population. This is done with different forecast time step lengths (i.e. 3, 6, 9, 12, 15 days). Below the dashed line, the within population forecasts are significantly ($p<0.05$) more skillful (i.e. lower MAE) than the between population forecasts.

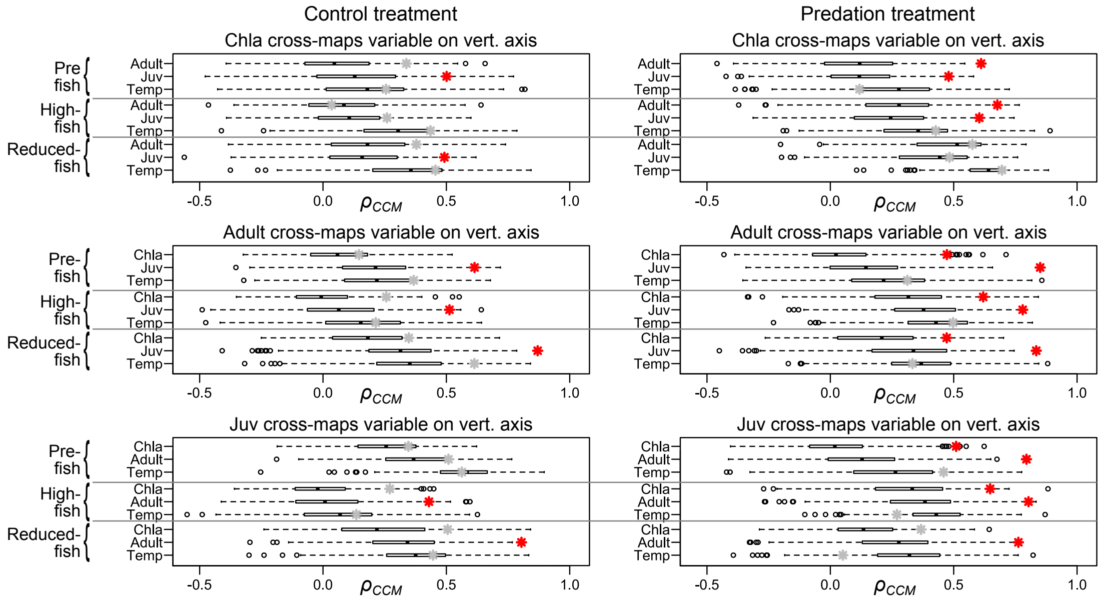

Figure SI9. Boxplots showing the null distributions of cross map skills (ρ_{ccm}) in the surrogate time-series for chlorophyll *a* concentration, the density of *Daphnia* adults and the density of *Daphnia* juveniles, for each population and both treatments separately. Cross map skills (ρ_{ccm}) in the original time-series are indicated with grey asterisks. Red asterisks indicate cross map skills (ρ_{ccm}) that were significantly larger than the surrogate time-series. The bottom and top of the box show the lower and upper quartiles, the band in between them shows the median, whiskers show the minimum and maximum (excluding outliers) and circles show the outliers. Outliers are values more than 1.5 times the length of interquartile range greater than the upper quartile or smaller than the lower quartile.

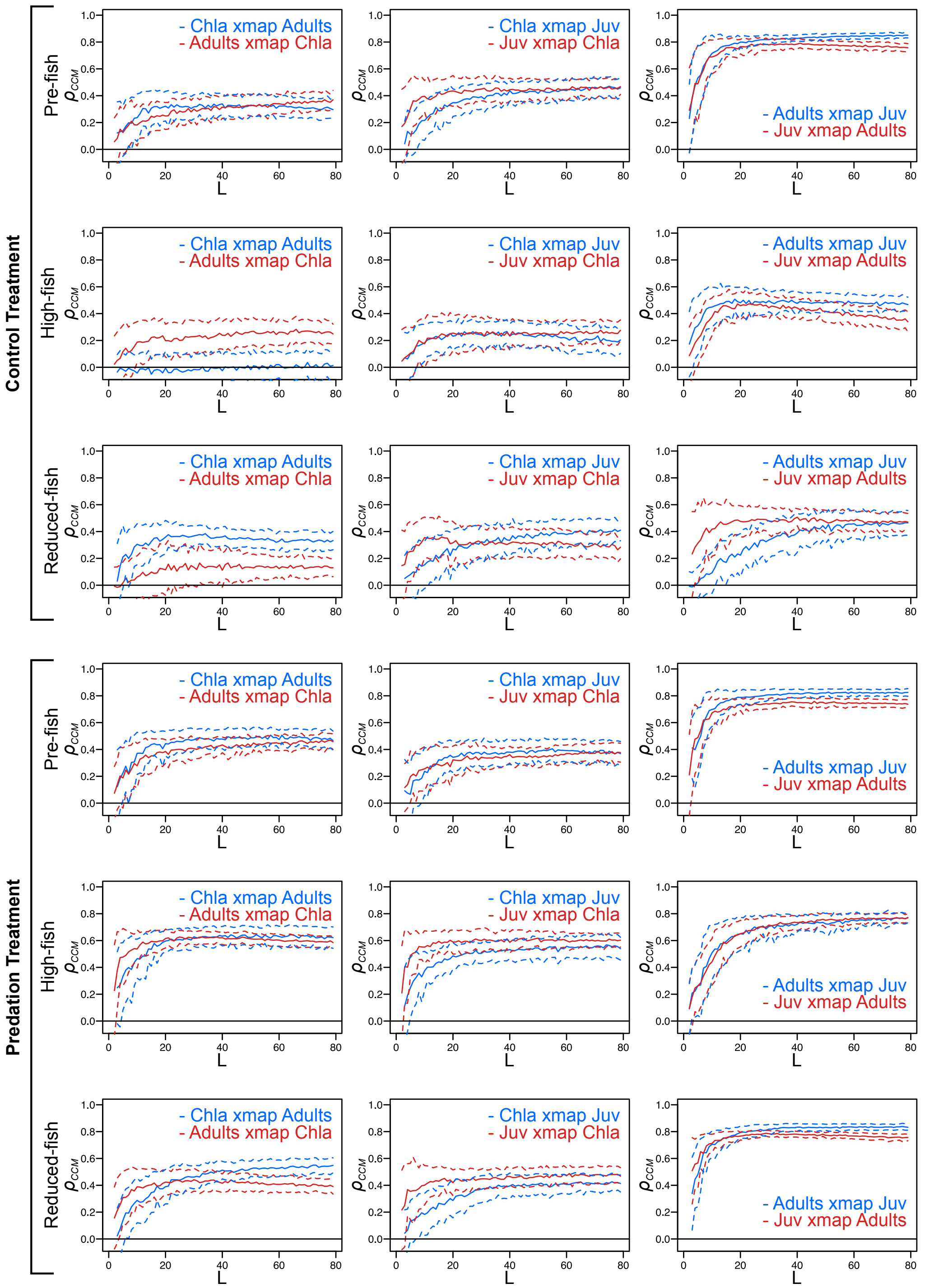

Figure SI10. Convergent cross-maps for each population in both treatments. Each plot shows the cross map between two variables in both directions against the length of the library set used to make the cross map. At each library length 100 random samples were used to make cross maps. The solid line shows the average cross map skill (ρ_{ccm}) and the dashed line shows the standard deviation. If variable X influences variable Y, the skill of the cross map from Y to X should initially increase and then converge to an upper limit, as the length of the library set used is increased.

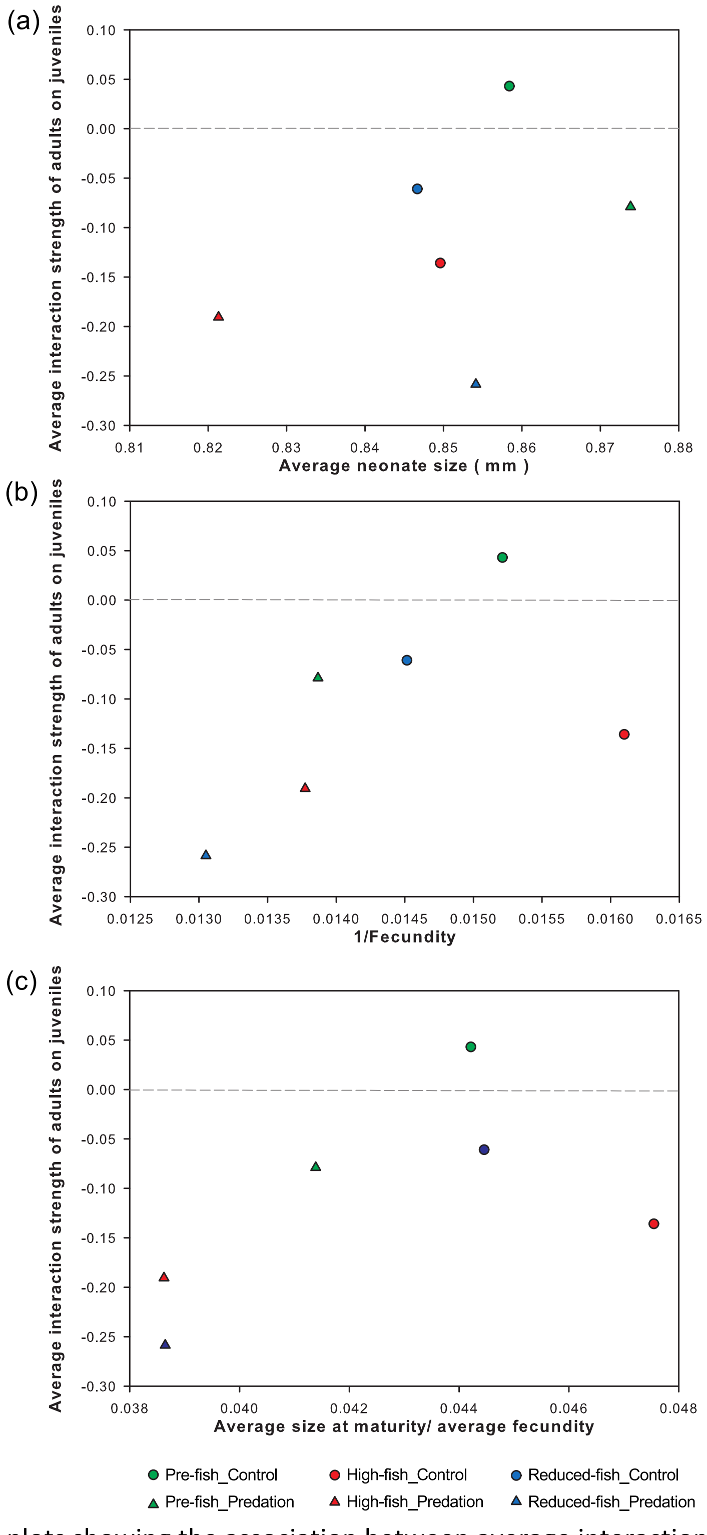

Figure SI11. Scatter plots showing the association between average interaction strengths of adults on juveniles against three measures of juvenile 'quality' based on life history data (Stoks et al., 2016): (a) average neonate size (body length); (b) inverse average number of offspring of the first two clutches (1/average fecundity); (c) average size at maturity divided by average fecundity.

References

Chang, C. W., Ushio, M., & Hsieh, C. H. (2017). Empirical dynamic modeling for beginners. *Ecological Research*. https://doi.org/10.1007/s11284-017-1469-9

Clark, A. T., Ye, H., Isbell, F., Deyle, E. R., Cowles, J., Tilman, G. D., & Sugihara, G. (2015). Spatial convergent cross mapping to detect causal relationships from short time series. *Ecology, 96*(5), 1174-1181.

Deyle, E. R, Maher, M. C., Hernandez, R. D., Basu, S., & Sugihara, G. (2016). Global environmental drivers of influenza. *Proceedings of the National Academy of Sciences*, 201607747.

Deyle, E. R., May, R. M., Munch, S. B, & Sugihara, G. (2016). Tracking and forecasting ecosystem interactions in real time. *Proceedings of the Royal Society B: Biological Sciences, 283*(1822).

Deyle, E. R., & Sugihara, G. (2011). Generalized theorems for nonlinear state space reconstruction. *PLoS One, 6*(3), e18295.

Hsieh, C. H., Anderson, C., & Sugihara, G. (2007). Extending nonlinear analysis to short ecological time series. *The American naturalist, 171*(1), 71-80.

Jansen, B., Geldof, S., De Meester, L., & Orsini, L. (2011). Isolation and characterization of microsatellite markers in the waterflea Daphnia magna. *Molecular Ecology Resources, 11*, 418-421.

Mergeay, J, Aguilera, X., Declerck, S., Petrusek, A., Huyse, T., & De Meester, L. (2008). The genetic legacy of polyploid Bolivian Daphnia: the tropical Andes as a source for the North and South American D. pulicaria complex. *Molecular Ecology, 17*(7), 1789-1800.

Orsini, L., Spanier, K. I, & De Meester, L. (2012). Genomic signature of natural and anthropogenic stress in wild populations of the waterflea Daphnia magna: validation in space, time and experimental evolution. *Molecular Ecology, 21*(9), 2160-2175.

Pauwels, L., Stoks, R., Decaestecker, E., & De Meester, L. (2007). Evolution of heat shock protein expression in a natural population of Daphnia magna. *The American naturalist, 170*(5), 800-805.

Stoks, R., Govaert, L., Pauwels, K, Jansen, B., & De Meester, L. (2016). Resurrecting complexity: the interplay of plasticity and rapid evolution in the multiple trait response to strong changes in predation pressure in the water flea Daphnia magna. *Ecology Letters, 19*(2), 180-190.

Sugihara, G. (1994). Nonlinear forecasting for the classification of natural time series. *Philosophical Transactions of the Royal Society of London A: Mathematical, Physical and Engineering Sciences, 348*(1688), 477-495.

Sugihara, G., & May, R. M. (1990). Nonlinear forecasting as a way of distinguishing chaos from measurement error in time series. *Nature, 344*(6268), 734-741.

Sugihara, G., May, R., Ye, H., Hsieh, C., Deyle, E., Fogarty, M., & Munch, S. (2012). Detecting causality in complex ecosystems. *Science, 338*(6106), 496-500.

Takens, F. (1981). Detecting strange attractors in turbulence *Dynamical systems and turbulence, Warwick 1980* (pp. 366-381): Springer.

Ye, H., Clark, A., Deyele, E., Keyes, O. , & Sugihara, G. (2016). rEDM: Applications of Emeperical Dynamic Modelling from Time Series (Version 0.5.4). Retrieved from https://CRAN.R-project.org/package=rEDM
